# Developmental overproduction of cortical superficial neurons impairs adult auditory cortical processing

**DOI:** 10.1038/s41598-025-95968-x

**Published:** 2025-04-08

**Authors:** Mirna Merkler, Nancy Y. Ip, Shuzo Sakata

**Affiliations:** 1 Strathclyde Institute of Pharmacy and Biomedical Sciences, University of Strathclyde, 161 Cathedral Street, Glasgow, G4 0RE UK; 2 Division of Life Science, State Key Laboratory of Molecular Neuroscience and Molecular Neuroscience Center, The Hong Kong University of Science and Technology, Clear Water Bay, Kowloon, Hong Kong China

**Keywords:** Auditory cortex, Cortical layers, Perceptual decision, Neurodevelopmental disorders, Autism spectrum disorder, Neuropixels, Neural circuits, Cortex

## Abstract

While evolutionary cortical expansion is thought to underlie the evolution of human cognitive capabilities, excessive developmental expansion can lead to megalencephaly, often found in neurodevelopmental disorders. Still, little is known about how the overproduction of cortical neurons during development affects cortical processing and behavior in later life. Here we show that developmental overproduction of cortical superficial neurons impairs auditory processing in adult mice. We applied XAV939 to overproduce cortical superficial excitatory neurons during development. XAV939-treated adult mice exhibited auditory behavioral deficits and abnormal auditory cortical processing. Furthermore, we found fewer functional monosynaptic connections between cortical putative excitatory neurons. Altogether, our results suggest that abnormal auditory cortical processing contributes to the atypical auditory detectability in XAV939-treated mice. Although the expansion of cortical size is evolutionarily advantageous, an abnormal expansion during development can result in detrimental effects on cortical processing and perceptual behavior in adulthood.

**Supplementary Information:**

The online version contains supplementary material available at 10.1038/s41598-025-95968-x.

## Introduction

The mammalian brain has evolved the neocortex with a prominent six-layered structure^1–3^. Over a hundred transcriptionally distinct neuron types can be identified across cortical laminae and they exhibit highly diverse functional properties^4–9^. Although we have witnessed tremendous progress on the characterization of neuronal diversity and circuit operations in the neocortex over the past decade, neuronal number has also long been considered a crucial factor for neural function^10,11^. For instance, the number of active neurons has been recognized as a fundamental feature to realize efficient neuronal processing^12,13^. Nonetheless, we still know little about to what extent the changes in the number of cortical neurons affect neural computation.

The number of cortical neurons is primarily determined by two fundamental factors in development, that is, the length of the neurogenic period and the number of neurons generated per unit of time^14,15^. Deficits in neural stem cell proliferation and differentiation can lead to neurodevelopmental disorders, such as autism spectrum disorder (ASD) and intellectual disability^16,17^. While a wide range of anatomical abnormalities has been associated with ASD, macrocephaly can be observed in ~ 15% of autistic patients^18,19^. However, it remains unclear how such abnormalities in cortical development can lead to abnormalities in cortical information processing and behavior in later life.

Over the past decade, various mouse models of neurodevelopmental disorders have been developed^20,21^. While some models reflect genetic deficits in human patients, environmental factors during development can also contribute to ASD^22,23^. Yet, it is still challenging to manipulate developmental processes, especially the number of neurons, in a time-limited fashion.

In the present study, we utilize a small molecule, XAV939, which inhibits Tankyrase and results in Axin stabilization by preventing its degradation through the ubiquitin-proteasome pathway^24^. Axin, a scaffolding protein crucial for cortical development^25,26^, contains several functional domains to interact with signaling molecules, including Tankyrase. When Axin is ADP-ribosylated by Tankyrase, the ubiquitin-proteasome system recognizes it to breakdown. Therefore, inhibiting Tankyrase with XAV939 leads to the accumulation of Axin complex. Since the Axin complex promotes β-catenin degradation^27^, XAV939 primarily acts as a Wnt/β-catenin pathway inhibitor^24^. Notably, mutations in the *Axin* gene are associated with abnormal brain size^28,29^.

Due to these effects, in utero microinjections of XAV939 in mouse embryos at embryonic day 14.5 elevate Axin levels and transiently amplify intermediate progenitors, resulting in the overproduction of cortical superficial excitatory neurons, without affecting the production of interneurons, microglia or astrocytes, or the number of hippocampal neurons^25,30^. While these increased cortical superficial neurons are maintained in adults, several age-dependent changes are observed in this XAV939 model^30^. First, although a higher density of mature spines is seen at postnatal day (P) 18, spine density significantly decreases by P60. Second, consistent with these morphological changes, although larger field excitatory post-synaptic potentials (fEPSPs) are observed at P18 compared to control, fEPSPs become smaller by P60. However, the impact of these changes on neural population activity and behavior remains to be explored.

At the behavioral level, this XAV939 model develops autism-like behavioral phenotypes, such as excessive grooming and impairments in social behaviors^30^. This model also forms more neuronal ensembles in visual cortical layer L2/3 and demonstrate improved visual acuity^31^. While ASD patients often exhibit abnormal sensitivity to acoustic stimuli^32,33^, it is unknown if this mouse model develops an abnormal detectability to sounds. By combining in vivo electrophysiological and behavioral approaches, we address this issue. Here we report XAV939-induced hyposensitivity to acoustic stimuli, manifesting through reduced behavioral auditory detection as well as altered neuronal activity in the auditory cortex.

## Results

### Overproduction of cortical superficial neurons by in utero XAV939 microinjections

**Fig. 1:**
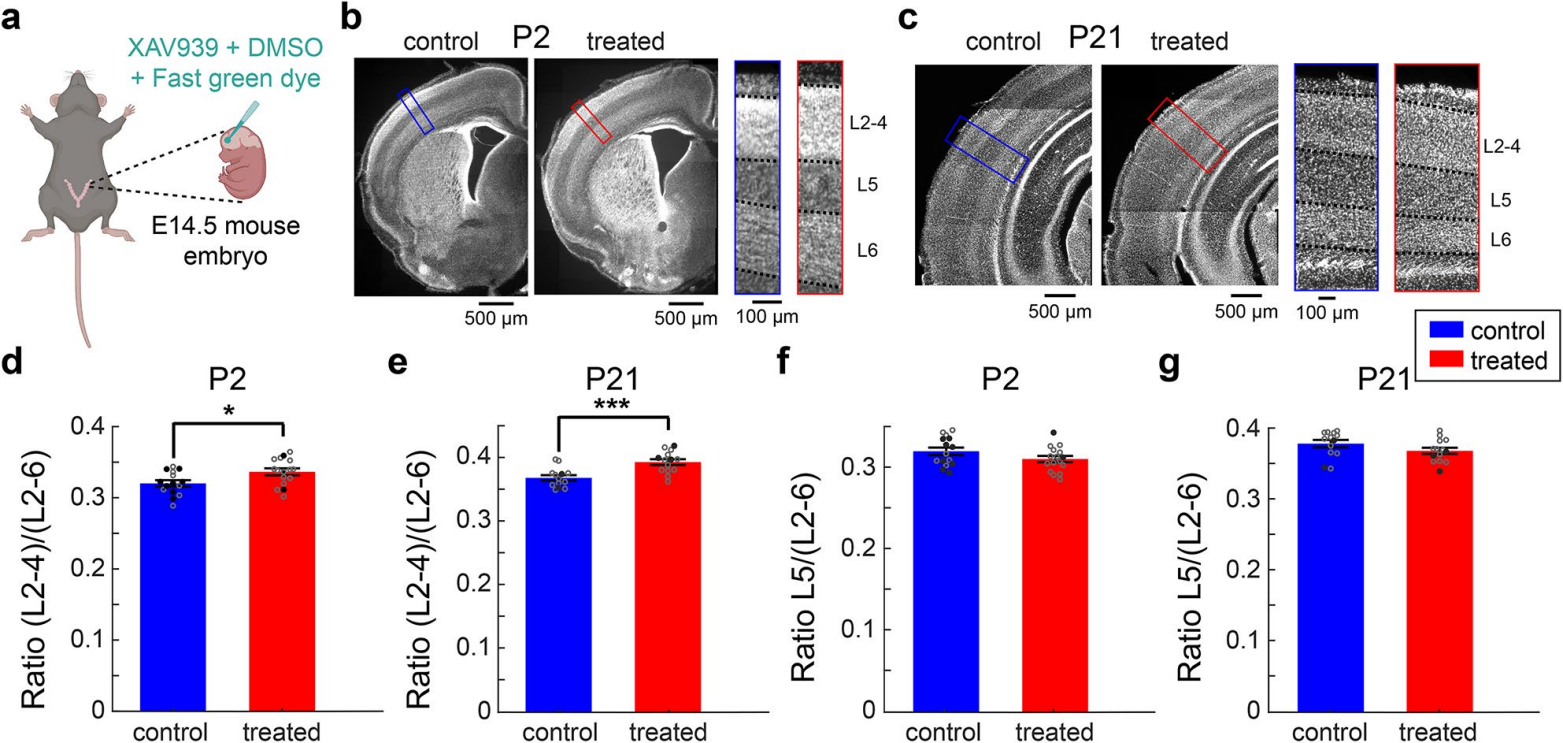
XAV939 caused an increase in the width of cortical superficial layers. (**a**) Schematics of *in utero* microinjections. Image was created in BioRender. Merkler, M. (2025) https://BioRender.com/i45p356. (**b** and **c**) Comparison of cortical layer width between a control and a treated mouse on postnatal day 2 (P2) (**b**) and P21 (**c**), with the corresponding magnified cortical areas. (**d** and **e**) Fraction of cortical superficial layers (L2-4) at P2 (**c**) and P21 (**d**). (**f** and **g**) Fraction of L5 at P2 (**f**) and P21 (**g**). Error bars indicate SEM; filled black scatter points indicate males, empty grey indicate females; *p < 0.05, ***p < 0.001; *t*-test (**d–g**).

To overproduce cortical superficial neurons, we injected XAV939 into the lateral ventricle of mouse embryos at embryonic day (E) 14.5, when intermediate progenitor cells that will differentiate into L2/3 excitatory neurons are proliferating (Fig. 1a). To examine the effect of XAV939 microinjections, we performed histological analysis at postnatal day (P) 2 and 21 (Figs. 1b–g and S1). As reported previously^30^, we confirmed that cortical superficial layers are significantly expanded in treated animals compared to vehicle controls at P2 (*n*_control_ = 15 animals, *n*_treated_ = 16 animals, *p* < 0.05, *t*-test) (Fig. 1b, d) and P21 (*n*_control_ = 13 animals, *n*_treated_ = 14 animals, *p* < 0.001, *t*-test) (Fig. 1c, e). In adult (> P60) mice, we saw the trend for increased superficial layers, as observed in younger animals (*n*_control_ = 6 animals, *n*_treated_ = 8 animals, *p* = 0.06, *t*-test; Hedge’s *g* = -1.03) (Fig. S5c). Consistent with previous reports^25^, where the number of Ctip2 + cells in deeper cortical layers was slightly but non-significantly reduced in XAV939 mice, we also observed a slight, non-significant reduction in L5 width (*p*_*P2*_ = 0.12, *p*_*P21*_ = 0.14, *t*-test) (Fig. 1b, c, f and g). When we looked at the effect of drug volume, we saw that even with a smaller amount the drug effect on layer width at P2 (*n*_*low*_ = 9 animals, *n*_*high*_ = 7 animals) or P21 (*n*_*low*_ = 6 animals, *n*_*high*_ = 8 animals) is consistent (*p*_*P2*_ = 0.1 and *p*_*P21*_= 0.7, *t*-test) (Fig. S5a). Using this animal model, we investigated whether and how the developmental overproduction of cortical superficial neurons affects auditory processing in adults.

### Hyposensitivity to auditory stimulus in XAV939-treated adult mice

**Fig. 2:**
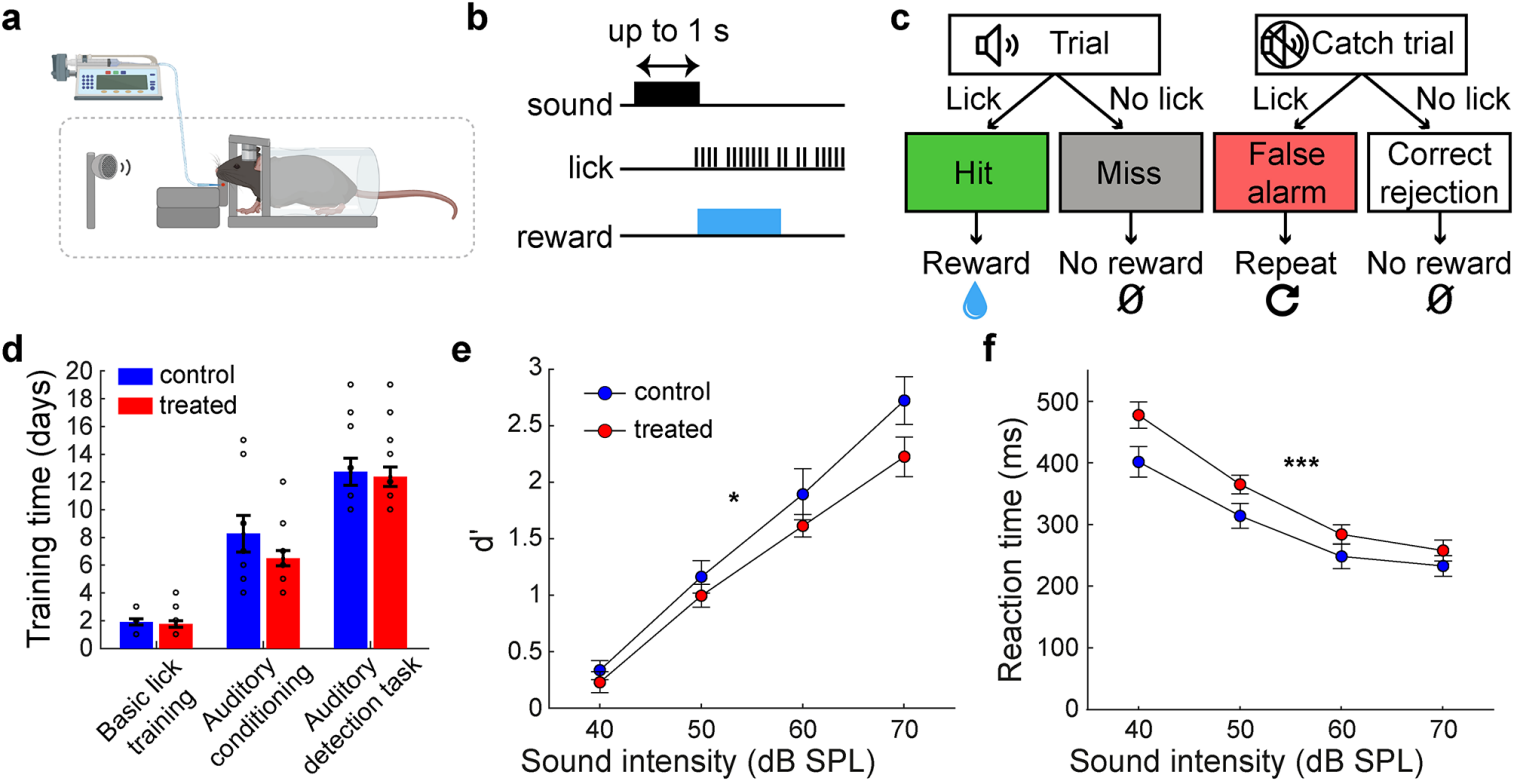
XAV939 treated mice exhibit auditory response deficits. (**a**) Schematics of head-fixed behavioral training. Elements located inside a soundproof box are positioned within the dashed box. Image was created in BioRender. Merkler, M. (2025) https://BioRender.com/a37u531. (**b**) Timeline of events in the auditory detection task. Trial starts with the sound onset. If the mouse licks during the response window (lasting 1 s), the sound turns off and water reward is delivered. (**c**) Diagram of possible events and outcomes during an auditory detection task session. (**d**) Comparison of training time the mice needed to pass each of the 3 phases in the behavioral assessment. (**e**) Comparison of *d’* between control and treated mice across sound intensities (*n* = 11 vs. 16 mice; 1 session per mouse). (**f**) Comparison of reaction time between control and treated mice across sound intensities (*n* = 11 vs. 16 mice; 1 session per mouse). Error bars indicate SEM; *p < 0.05, **p < 0.01; *t*-test (**d**), two-way ANOVA (**e** and **f**).

To examine the perceptual ability of XAV939-treated mice, we trained adult (~ P70) mice to perform an auditory detection task in a head-restrained condition (Fig. 2a). In this task, mice were required to perform a licking response when they perceived a broadband white noise with varied intensities. When mice correctly detected a sound, they were rewarded with water. In a “catch” trial, no sound was presented (Fig. 2b, c). Although both XAV939-treated and control mice passed all three pre-training phases equally (*p* = 0.63 for basic lick training, *p* = 0.1761 for auditory conditioning, *p* = 0.76 for auditory detection, *t*-test) (Fig. 2d), we found a significant effect of treatment on detection performance, measured by detectability index *d’* (*n*_control_ = 11, *n*_treated_ = 16, *F*_1,100_ = 6.48, *p* < 0.05, two-way ANOVA; *d’* = *Z*(hit rate) - *Z*(false alarm rate)) (Fig. 2e). We also estimated the detection threshold by applying a logistic regression analysis (see Materials and Methods). We found a trend of the higher detection threshold in XAV939-treated mice compared to control mice (*n*_control_ = 11, *n*_treated_ = 16, *p* = 0.06, rank sum test) (Fig. S2). Consistent with these observations, the effect of treatment on the average reaction time was also significant (*n*_control_ = 11, *n*_treated_ = 16, *F*_1,100_ = 12, *p* < 0.001, two-way ANOVA) (Fig. 2f). We also compared the number of behavioral trials between the groups. We found no significant difference in the number of trials (*n*_control_ = 11, *n*_treated_ = 16, *p* = 0.62, rank sum test) (Fig. S6). We further looked at the drug volume effect on behavioral outcomes, and we found no significant differences in d’ (*n*_low_ = 6, *n*_high_ = 10, *F*_1,56_ = 42.47, *p* = 0.8, two-way ANOVA) or reaction time (*n*_low_ = 6, *n*_high_ = 10, *F*_1,56_ = 36.12, *p* = 0.1, two-way ANOVA) (Fig. S5b) between low and high volumes of XAV939. Thus, the developmental overproduction of cortical superficial neurons led to a hyposensitivity to auditory stimulus in adult mice.

### Abnormal spontaneous population activity in XAV939-treated auditory cortex

**Fig. 3:**
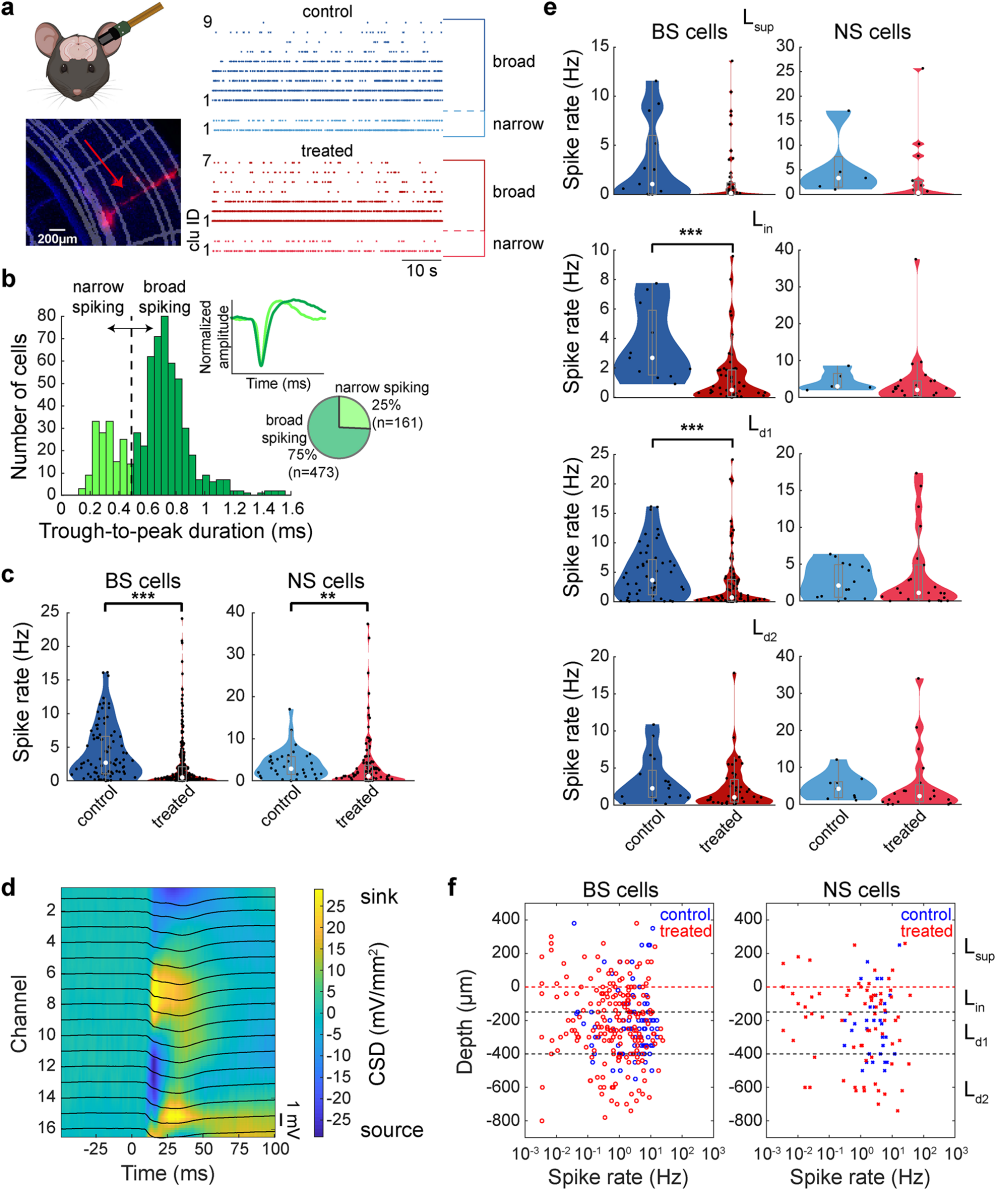
Spontaneous cortical activity was significantly altered in treated mice. (**a**) Schematics of Neuropixels probe insertion (top left) and histological confirmation of the probe location in the auditory cortex (bottom left). The schematics was created in BioRender. Merkler (2025) https://BioRender.com/a38v236. Example raw traces of spontaneous firing of auditory cortical single-unit clusters in a control and a treated mouse (right). Clusters were divided into broad-spiking and narrow-spiking. (**b**) Cell type classification depending on their waveform trough to peak duration. Two clusters (broad-spiking; BS and narrow-spiking; NS) were split at 0.5 ms border. Top inset: comparison between narrow and broad waveform. Bottom inset: Total number and percentages of BS and NS neurons across all recordings (passive listening and task performing), used in further analysis. (**c**) Spontaneous activity in control and treated mice (*n* = 89 vs. 281 BS cells, and 34 vs. 101 NS cells). (**d**) Current source density (CSD; colormap) and event-related potential (ERP; black traces) example for one shank (16 channels) of a silicon probe recording. Top current sink channel (yellow; channel 8) is set as depth zero and the top border of input layer (L_in_). Using CSD, neurons were divided into cortical layers depending on the depth of the channel with their largest amplitude of average spike waveforms. (**e**) Spontaneous activity across cortical layers. L_sup_, putative L2/3; L_in_, putative L4; L_d1_, putative L5; L_d2_, putative L6. (**f**) Scatter plots of BS (left) and NS (right) neurons, showing their depth and cortical layer position as well as their spontaneous activity. Estimated layer borders are as follows: Superficial layers (L_sup_) > 0 μm, input layer (L_in_) 0 to -150 μm, deep layer 1 (L_d1_) -150 to -400 μm, deep layer 2 (L_d2_) < -400 μm. **p < 0.01, ***p < 0.001; Rank sum test (**c** and **e**).

**Fig. 4:**
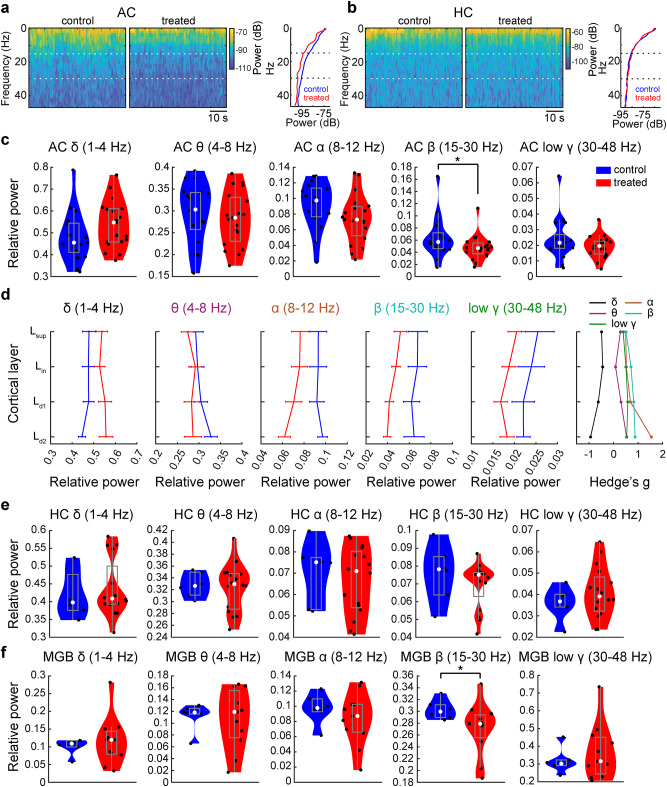
Local field potential changes in treated mice were confined to the cortex. (**a**) Power spectrum density (PSD) of LFP signals in the auditory cortex during silent period in a control and a treated mouse (left). Plots show the mean power across frequencies from the same mice (right). Beta frequency range (area between the dotted lines) shows frequency-specific treatment-related differences between the two animals. (**b**) PSD of LFP signals in the hippocampus during silent period in a control and a treated mouse (left). Mean power across frequencies plots from the same mice (right). (**c**) Comparison of relative power across frequency bands in the auditory cortex of control and treated mice during silence. Mean relative power was calculated across cortical channels for each recording (*n* = 17 vs. 19 recordings). (**d**) Relative resting state power across cortical layers, for each frequency band. Hedge’s *g* was calculated to look at the effect of treatment for each cortical layer across frequency bands. (**e**) Mean relative power across hippocampal channels during silence, for each frequency band (*n* = 6 vs. 16 recordings). (**f**) Relative resting state power of MGB neuronal spike trains, across frequency band (*n* = 6 vs. 11 recordings). Error bars indicate SEM; *p < 0.05; rank sum test (c, e and f).

**Fig. 5:**
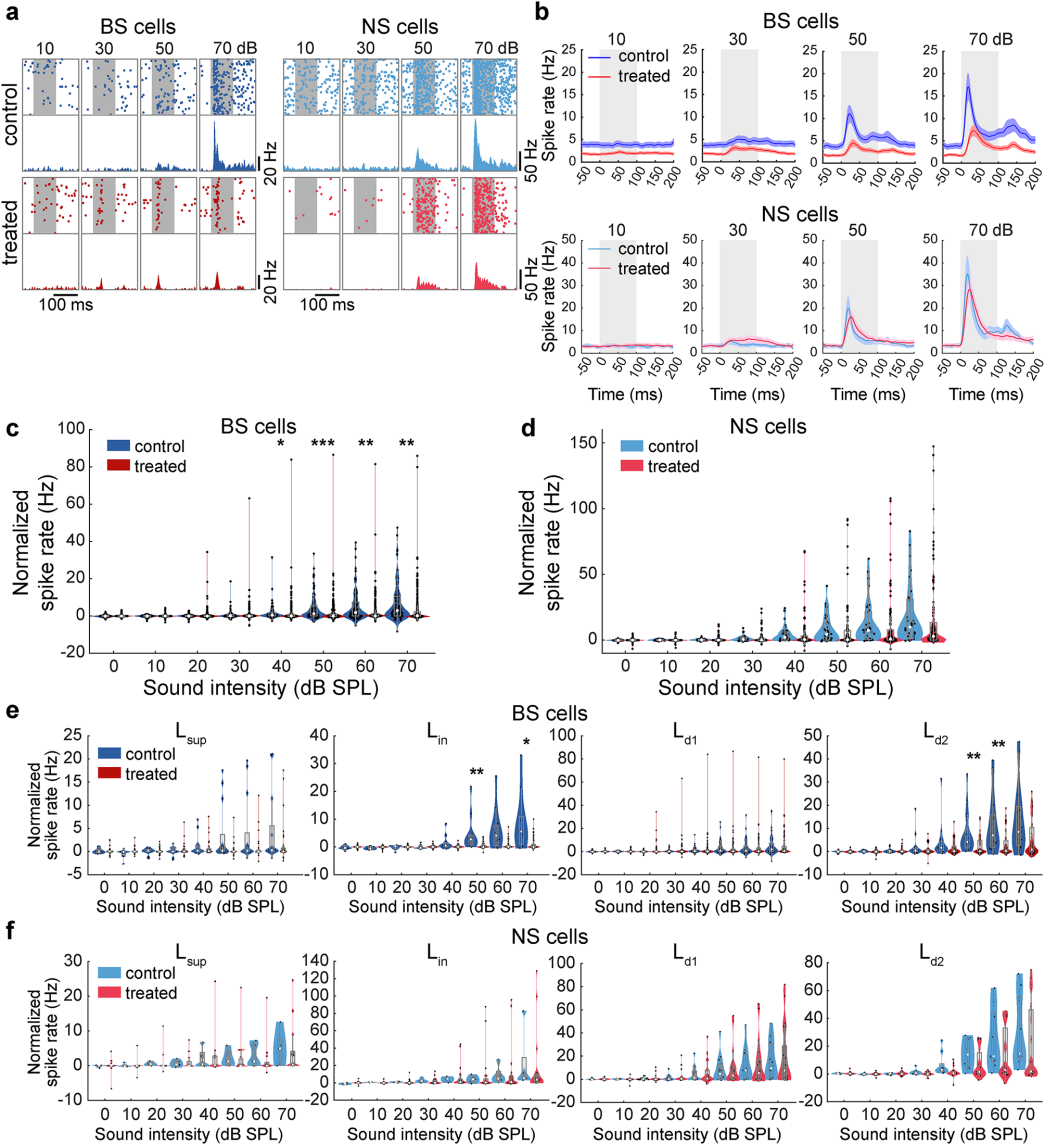
Auditory evoked activity was affected by XAV939 treatment. (**a**) Scatter plot and histogram of activity of one BS (left) and one NS (right) neuron of a control (top) and a treated (bottom) mouse, across sound intensities (100 repetitions per intensity). Sound period is labelled as a gray background, lasting 100ms. (**b**) Peristimulus time histogram (PSTH) of activity (mean ± SEM) of BS neurons (top) and NS neurons (bottom) in control and treated group during sound presentation. Sound period is labelled as a gray background (lasting 100ms). (**c** and d) Spike rate normalized by baseline in BS (**c**) and NS (**d**) neurons during first 50 ms of sound presentation. (**e** and **f**) Activity of cells shown in (**c**) and (**d**) split into cortical layers. *p < 0.05, **p < 0.01, ***p < 0.001; Rank sum test with Bonferroni correction (**c–f**).

**Fig. 6:**
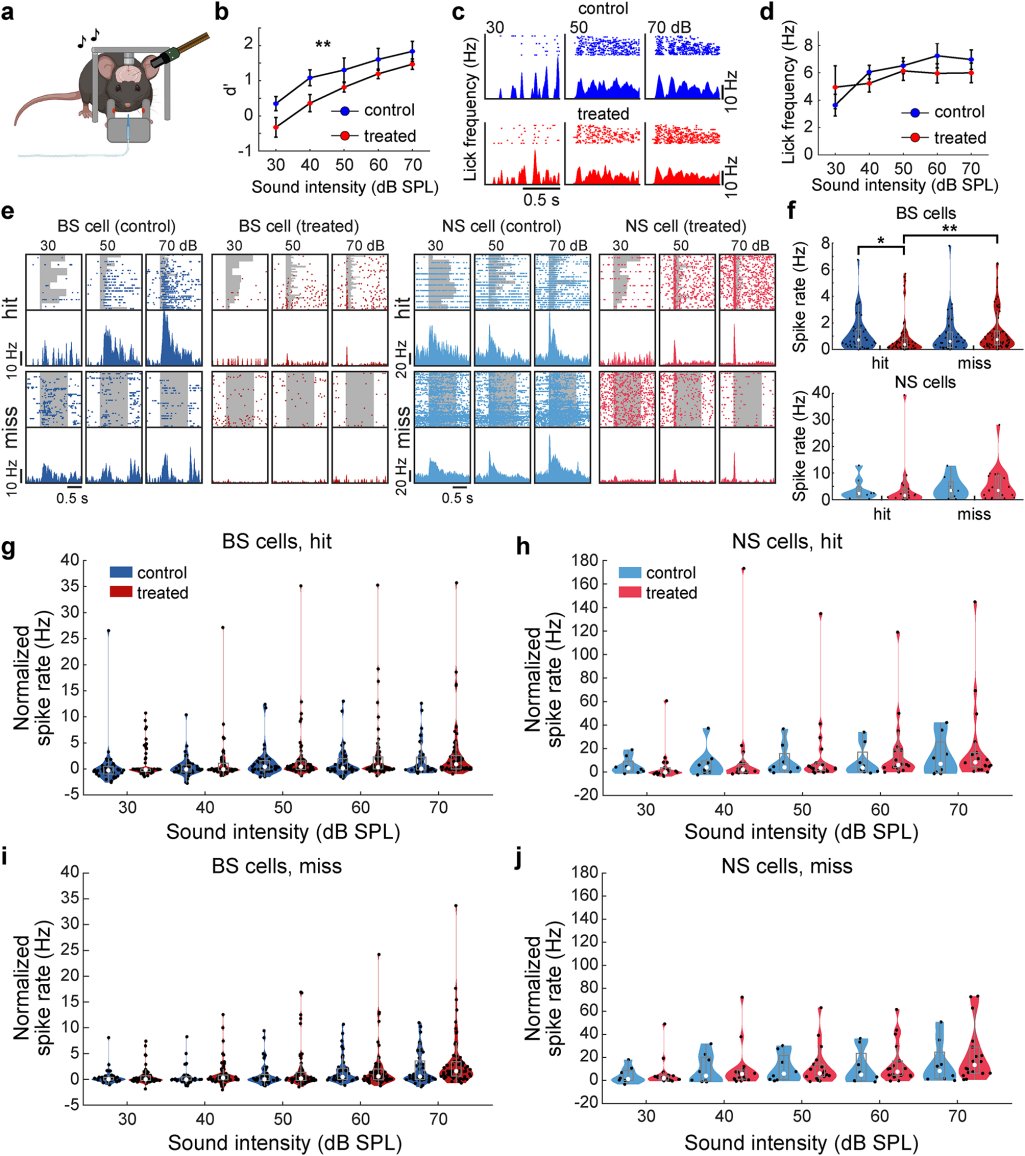
Trial outcome related differences in neuronal activity were observed in task behaving treated mice. (**a**) Schematics of Neuropixels recording in task behaving head-fixed conditions. Image was created in BioRender. Merkler (2025) https://BioRender.com/u76p325. (**b**) Comparison of *d’* between control and treated mice performing the task during electrophysiological recording, across sound intensities (*n* = 5 vs. 4 recordings). (**c**) Scatter plot and histogram of licking behavior during “hit” trials in a control and a treated mouse, across sound intensities. (**d**) Licking frequency comparison between control and treated mice, across sound intensities (*n* = 5 vs. 4 recordings). **(e)** Scatter plot and histogram of activity of one BS (left) and one NS (right) neuron of a control and a treated mouse, split into hit (top) and miss (bottom) trials, across sound intensities. Sound period is labelled as a gray background. (**f**) Spontaneous activity during prestimulus window (0.5 s before trial onset), before trials with “hit” or “miss” outcome (*n* = 40 vs. 63 BS cells, and 8 vs. 18 NS cells). (**g** and **h**) Activity of BS (**g**) and NS (**h**) neurons, normalized by baseline, in trials with “hit” outcome, during first 50 ms from sound onset. (**i** and **j**) Activity of BS (**i**) and NS **(j)** neurons, normalized by baseline, in trials with “miss” outcome. Error bars indicate SEM; *p < 0.05, **p < 0.01; two-way ANOVA (**b** and **d**), rank sum test (**f**), rank sum test with Bonferroni correction (**g–j**).

**Fig. 7:**
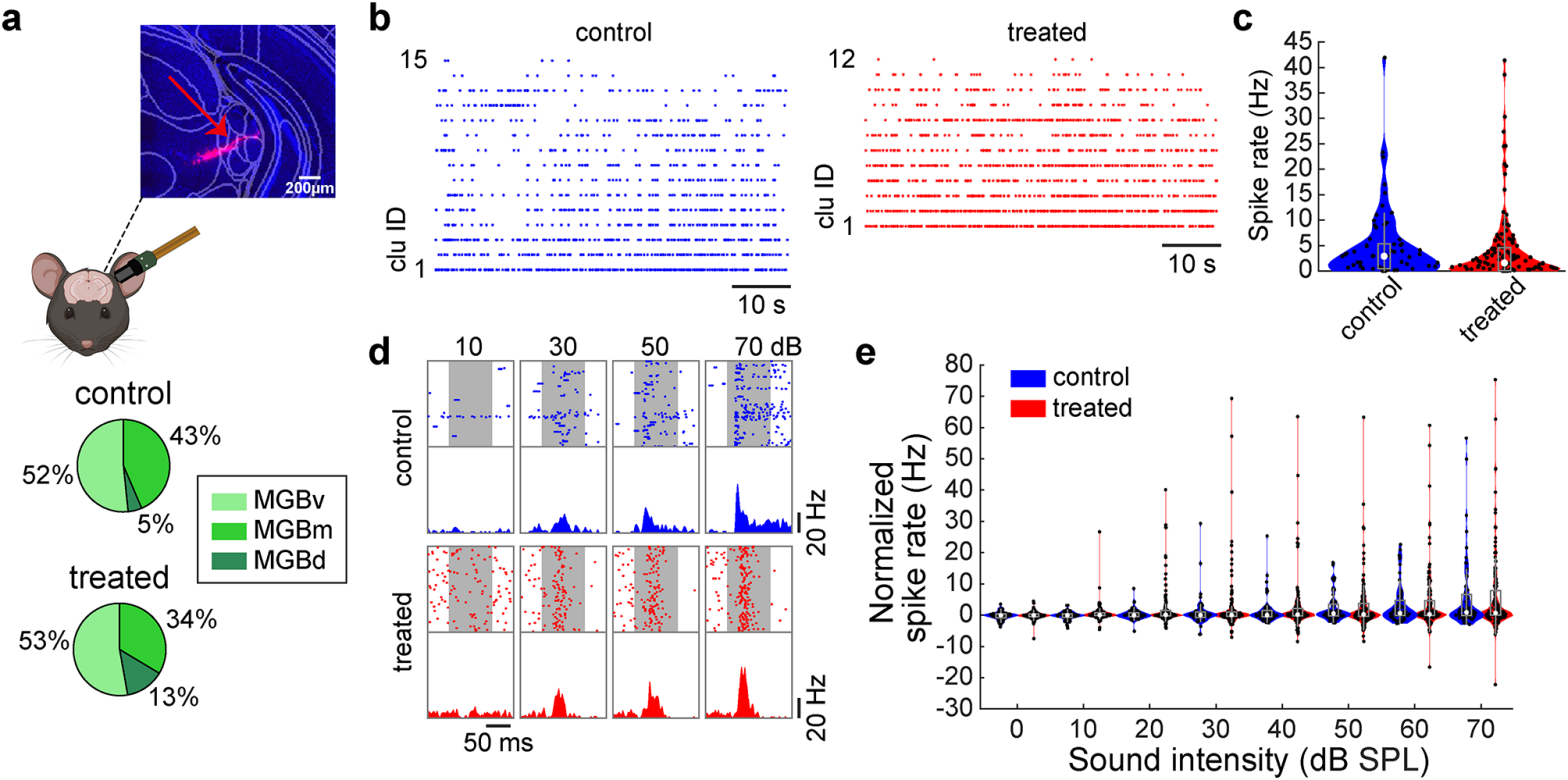
Auditory thalamic activity in passive listening mice was not affected by XAV939 treatment. (**a**) Schematics of simultaneous Neuropixels recording from auditory cortex (AC) and medial geniculate body (MGB), with histological confirmation of probe location in MGB (top). Percentage of MGB neurons recorded across different nuclei in control and treated mice (bottom). In our recordings, most of the neurons came from MGBv, with only a small fraction of MGBd neurons. The schematics was created in BioRender. Merkler, M. (2025) https://BioRender.com/a38v236. (**b**) Example raw traces of spontaneous firing of MGB single-unit clusters in a control (left) and a treated (right) mouse. (**c**) Spontaneous activity in MGB during passive listening conditions (*n* = 62 vs. 127 cells). (**d**) Scatter plot and histogram of activity of one MGB neuron of a control (top) and a treated (bottom) mouse, across sound intensities (100 repetitions per intensity). Sound period is labelled as a gray background, lasting 100ms. (**e**) MGB activity normalized by baseline in passive listening conditions across sound intensities. None were statistically significant (*p* > 0.1); Rank sum test (**c**), rank sum test with Bonferroni correction (**e**).

**Fig. 8:**
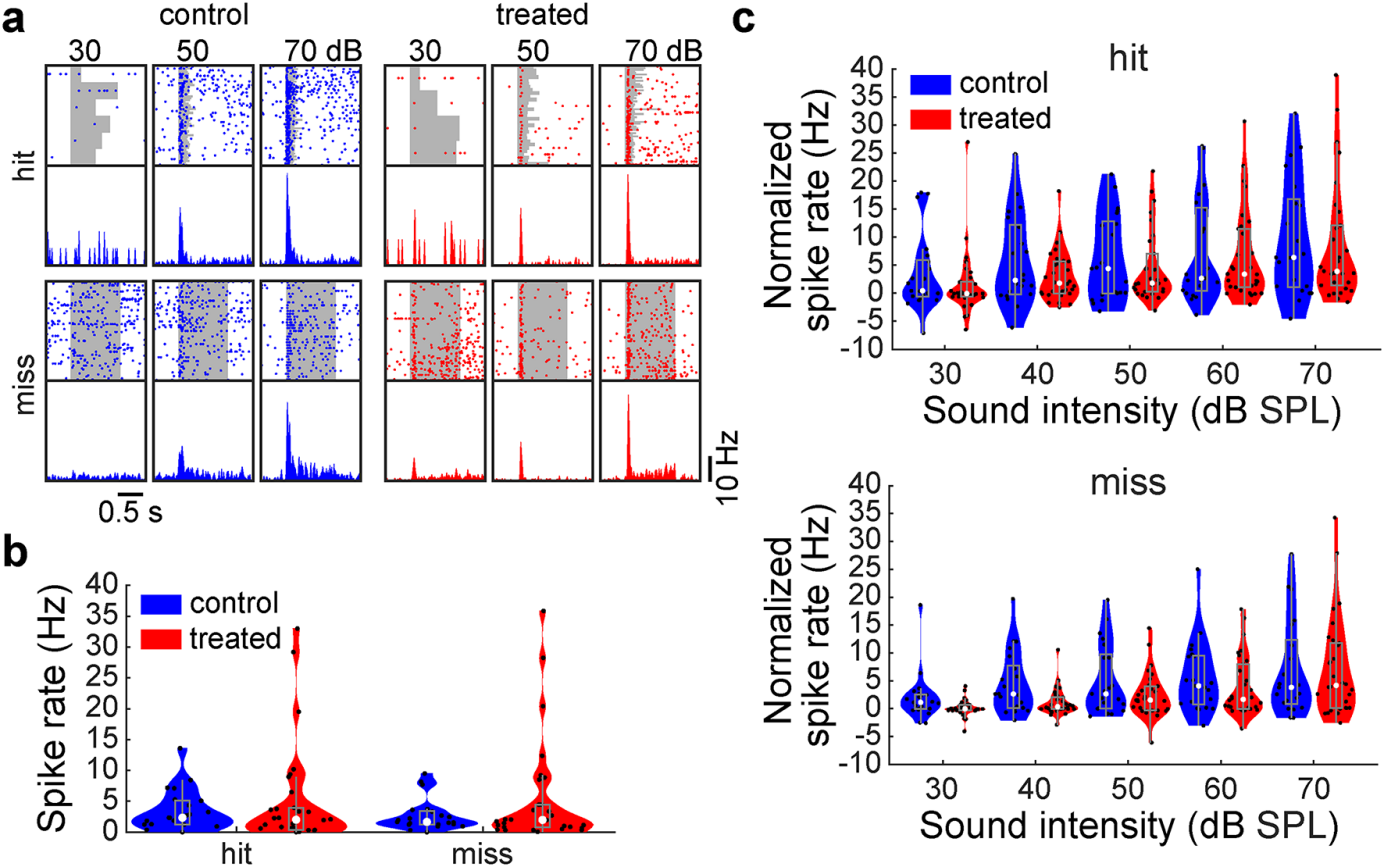
Auditory thalamic activity in task-performing mice was not affected by XAV939 treatment. (**a**) Scatter plot and histogram of activity of one MGB neuron in a control (top) and a treated (bottom) mouse, split into hit (left) and miss (right) trials, across sound intensities. A sound period is indicated as a gray background. (**b**) Spontaneous activity during the pre-stimulus window (0.5 s before trial onset), before trials with “hit” or “miss” outcomes (*n* = 21 vs. 30 cells). (**c**) MGB activity normalized by baseline in trials with “hit” (top) and “miss” (bottom) outcomes. None were statistically significant (*p* > 0.1); rank sum test (**b**), rank sum test with Bonferroni correction (**c**).

**Fig. 9:**
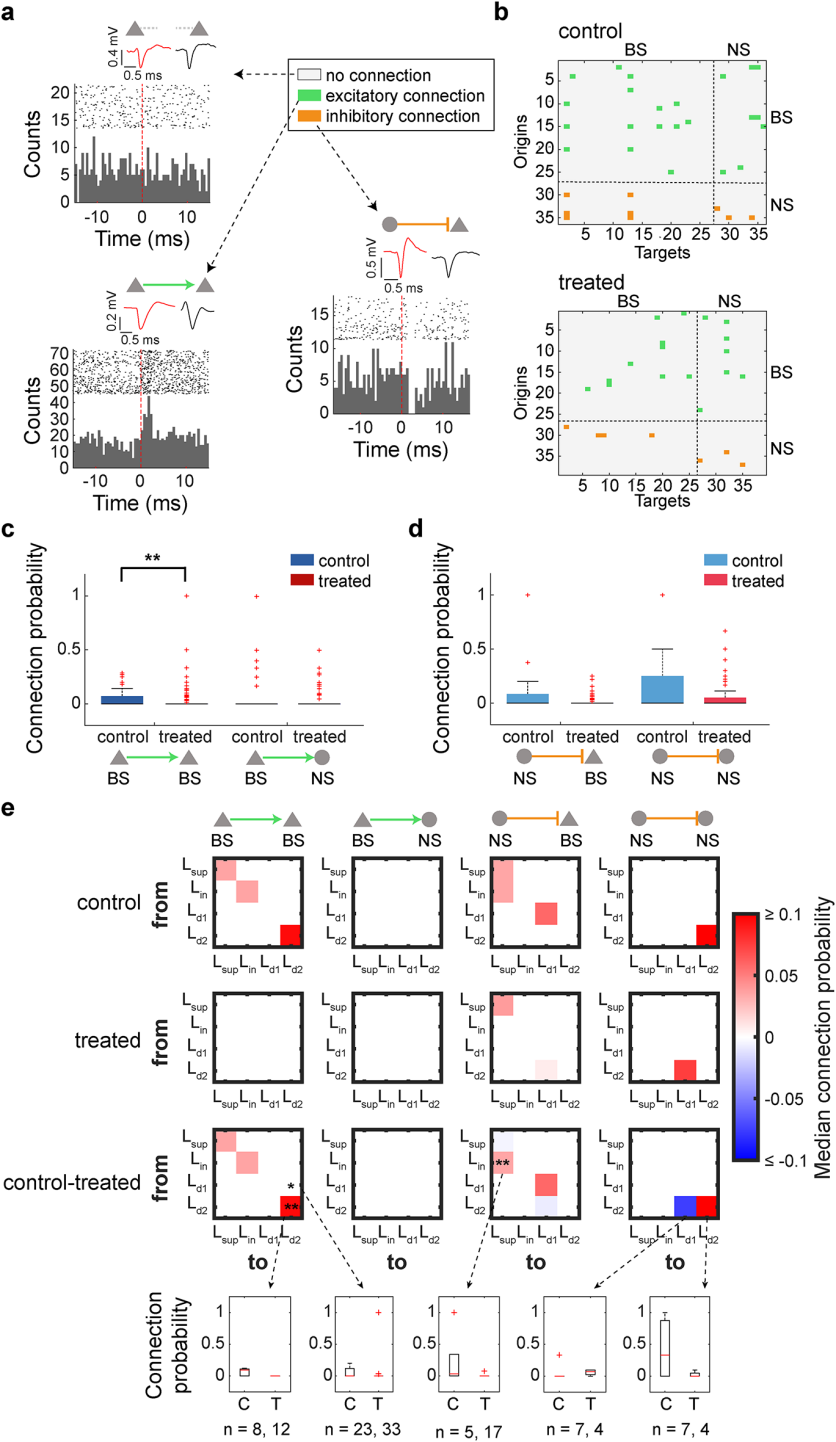
XAV939 treated mice have fewer monosynaptic connections within the auditory cortex. (**a**) Examples of cross-correlograms between neuron pairs; neurons that are not connected (top), excitatory connection (bottom left) and inhibitory connection (bottom right). Red dotted line marks the spike time of origin cell, while histogram shows the activity of targeted cell. Mean waveform of each example origin and targeted neuron is shown above their cross-correlogram. For these examples, 2000 random spike events of origin cell were taken. (**b**) Connectivity matrices from one control and one treated mouse, showing the connections between simultaneously recorded neuron pairs. (**c**) Probability of an excitatory connection coming from BS neurons (triangle; *n* = 129 vs. 340) to either BS (triangle) or NS neurons (circle). (**d**) Probability of an inhibitory connection coming from NS neurons (circle; *n* = 42 vs. 120). (**e**) Functional connectivity assessed across layers, for 4 different possible connections (BS-BS, BS-NS, NS-BS, NS-NS). In each matrix, rows represent the origin cortical layer (“from”), and columns represent the target cortical layer (“to”). The top row of matrices shows the median connection probabilities in cells from control animals, the second row shows probabilities in cells from treated animals, and the third row shows the difference between control and treated groups. Bottom row shows full connection probability comparison for specific examples (indicated by arrows). *p < 0.05, **p < 0.01; Rank sum test with Bonferroni correction (**c**,** d** and **e**).

To investigate the underlying neural mechanisms of the hyposensitivity to auditory stimulus, we performed in vivo electrophysiological recordings in both passive listening and task-performing conditions (Figs. 3, 4, 5, 6, 7, 8 and 9). We used either 64-channel silicon probes or Neuropixels probes to monitor population activity (Fig. 3a): the latter probes allowed monitoring activity from both the auditory cortex and the auditory thalamus simultaneously.

In the auditory cortex, we recorded from 463 neurons (382 from 13 passive listening animals across 24 recordings; 81 from 3 task-performing animals across 4 recordings) in treated animals and 171 neurons (123 from 10 passive listening animals across 18 recordings; 48 from 3 task-performing animals across 5 recordings) in control animals. In the auditory thalamus, we recorded from 157 neurons (127 from 6 passive listening animals across 11 recordings; 30 from 3 task-performing animals across 4 recordings) in treated animals and 83 neurons (62 from 3 passive listening animals across 6 recordings; 21 from 3 task-performing animals across 5 recordings) in control animals. We further classified cortical neurons into broad-spiking (BS; putative excitatory) neurons (*n*_control_ = 129; *n*_treated_ = 344) and narrow-spiking (NS; putative fast-spiking) neurons (*n*_control_ = 42; *n*_treated_ = 119) based on their spike waveforms (Fig. 3b).

Analyzing these datasets, we firstly examined whether and how spontaneous activity in the auditory cortex was modified in XAV939-treated animals under a passive listening condition (Fig. 3c and e). We found that the spontaneous firing rate of BS (*n*_control_ = 89 cells, *n*_treated_ = 281 cells, *p* < 0.001, rank sum test) (Fig. 3c, left), as well as NS neurons (*n*_control_ = 34 cells, *n*_treated_ = 101 cells, *p* < 0.01, rank sum test) (Fig. 3c, right) was significantly lower in treated animals compared to that in control animals. Based on the depth profile of spike waveforms and current source density (CSD) sink location (Fig. 3d), we broke down auditory cortical neurons according to their depths into superficial layers (L_sup_; putative L2/3), input layer (L_in_; putative L4), deep layer 1 (L_d1_; putative L5) and deep layer 2 (L_d2_; putative L6) (Figs. 3d and f, see also Materials and Methods). Across layers, we observed lower firing rate of BS neurons in treated mice, with significant difference in L_in_ and L_d1_ (*p* < 0.001, rank sum test) (Fig. 3e).

We also assessed spontaneous neural oscillations between groups based on local field potentials (LFPs) (Fig. 4). We noticed a trend of reduced beta (15–30 Hz) power in the auditory cortex of XAV939-treated mice (Fig. 4a). To quantify this, we computed relative power across frequency bands (Fig. 4c). We found that the beta band power was significantly reduced in treated animals (*p*_beta_ < 0.05; *n*_control_ = 17 recordings, *n*_treated_ = 19 recordings, rank sum test). In Fig. 4d, we also assessed LFP powers across cortical layers and frequency bands. The effect size (Hedge’s *g*) was consistently high (~ 1) across layers in beta band.

By taking the advantage of Neuropixels probe recording, we also assessed simultaneously monitored LFPs in the hippocampus and multiunit activity (MUA) in the medial geniculate body (MGB) to examine if similar changes are seen upstream, in the thalamic region of the auditory pathway. In the hippocampus (Fig. 4b and e), we did not find any significant differences in oscillatory powers between two animal groups across frequency bands (*n*_control_ = 6 recordings, *n*_treated_ = 16 recordings, *p*_delta_ = 0.48, *p*_theta_ = 0.85, *p*_alpha_ = 0.74, *p*_beta_ = 0.22, *p*_gamma_ = 0.48; rank sum test), suggesting that the changes in cortical LFPs were not due to volume conduction from the hippocampus. On the other hand, MUA in the MGB exhibited significant changes in beta band power between groups (*p*_beta_ < 0.05; *n*_control_ = 6 recordings, *n*_treated_ = 11 recordings, rank sum test) (Fig. 4f). Additionally, we examined the spike-field coherence between MGB and the auditory cortex. We found no significant differences in coherence between the groups across the frequency bands (*n*_control_ = 4 recordings, *n*_treated_ = 8 recordings, *F*_1,50_ = 0.54, *p* = 0.47, two-way ANOVA) (Fig. S4). These results indicate that the overall spiking activity of BS neurons reduces, and the power of cortical beta oscillations decreases in XAV939-treated animals along with MGB populations.

### Abnormal auditory-evoked activity in XAV939-treated auditory cortex

Because the behavioral detection was significantly reduced in the treated mice (Fig. 2e), we hypothesized that auditory evoked responses in the auditory cortex of the treated animals are diminished. To test this, we began by comparing auditory evoked responses in BS and NS cells in treated and control mice in a passive listening condition (Fig. 5). As the sound intensity increased, evoked responses became larger. However, we noticed that evoked responses of BS neurons in treated mice were consistently lower than those in control mice (Figs. 5a and b). To confirm this quantitatively, we computed the mean spike rate in a 50 ms window from the stimulus onset across cells by subtracting pre-stimulus baseline firing (Figs. 5c and d). In BS cells, auditory-evoked responses were significantly reduced in treated animals for 40–70 dB sound intensities (*n*_control_ = 89 cells, *n*_treated_ = 281 cells, *p*_40_ < 0.05, *p*_50_ < 0.001, *p*_60_ < 0.01, *p*_70_ < 0.01, rank sum test with Bonferroni correction) (Fig. 5c). NS neurons in treated mice tended to exhibit slightly lower auditory-evoked responses, but with no significance (*n*_control_ = 34 cells, *n*_treated_ = 101 cells, *p* > 0.05 across all intensities, rank sum test with Bonferroni correction) (Fig. 5d).

We further assessed the laminar specificity in firing rate changes (Fig. 5e and f). Evoked responses tended to be lower across layers, with significant reduction in L_in_ and L_d2_ BS neurons. On the other hand, NS cells showed somewhat different effects but generally followed a reduction trend in all layers, particularly at higher intensities, with no significant group differences (Fig. 5f). To address the overall sample size imbalance between treated and control groups, we performed resampling (Fig. S7). The probability of distribution overlap was significantly low in all cases except for evoked responses of BS neurons at 0–20 dB, and evoked responses of NS neurons at 0 dB and 30 dB (Table S1). Overall, XAV939-treatment led to a reduction in auditory evoked responses in BS neurons, but not NS neurons, across cortical layers.

### Abnormal auditory cortical activity in task-performing XAV939-treated mice

Next, we directly compared auditory cortical activity in task-performing animals between the two groups (Fig. 6a). We confirmed that this independent cohort of animals also exhibited reduced detectability of sounds in the XAV939-treated condition (*n*_control_ = 5 recordings, *n*_treated_ = 4 recordings, *F*_1,35_ = 12.27, *p* < 0.01, two-way ANOVA) (Fig. 6b) whereas licking behavior was comparable between groups (*n*_control_ = 5 recordings, *n*_treated_ = 4 recordings, *F*_1,35_ = 0.71, *p* = 0.40, two-way ANOVA) (Figs. 6c and d). As neural activity in a pre-stimulus period can influence behavioral performance^34^, we examined if the spontaneous activity in the pre-stimulus window differed between animal groups depending on their behavioral outcomes, i.e., “hit” and “miss” (Fig. 6e). In hit trials, we found significantly lower pre-stimulus spontaneous activity of BS neurons in XAV939-treated mice compared to control mice (*n*_control_ = 40, *n*_treated_ = 63, *p* < 0.05, rank sum test) (Fig. 6f). On the other hand, spontaneous activity in miss trials was not significantly different between the groups (*n*_control_ = 40, *n*_treated_ = 63, *p* = 0.15, rank sum test) (Fig. 6f). Although the spontaneous activity is similar between hit and miss trials in control mice (*p* = 0.56, rank sum test), the spontaneous activity in treated mice was significantly higher in miss trials compared to hit trials (*p* < 0.01, rank sum test). Despite these differences in BS cells across conditions, we did not observe significant changes in spontaneous activity of NS neurons regardless of the outcome (*n*_control_ = 8, *n*_treated_ = 18, *p*_hit_ = 0.58, *p*_miss_ = 0.14, rank sum test) (Fig. 6f).

We also looked at the auditory evoked responses in each group for hit and miss trials by subtracting pre-stimulus spontaneous activity. The activity of BS and NS neurons did not exhibit any significant changes or trends in activity regardless of trial outcome (BS: *n*_control_ = 40, *n*_treated_ = 63, *p* > 0.05, across all intensities, NS: *n*_control_ = 8, *n*_treated_ = 18, *p* > 0.05, across all intensities, rank sum test with Bonferroni correction) (Fig. 6g–j). Thus, the poor detection performance of XAV939-treated mice was associated with a bigger change in pre-stimulus activity of BS neurons, rather than auditory evoked activity.

### Auditory thalamic activity in XAV939-treated mice

To examine whether the observed abnormal activity in the auditory cortex of XAV939-treated animals is inherited from an upstream structure, we analyzed activity in the MGB of passive listening (Fig. 7). Neurons were recorded from multiple auditory thalamic nuclei, not just the ventral division of the medial geniculate body (MGBv) (Fig. 7a). As we did for auditory cortical neurons, we began by qualitatively comparing MGB neurons between animal groups with respect to their spontaneous activity (Fig. 7b) and auditory evoked activity (Fig. 7d). We found that MGB neurons did not show any significant differences in spontaneous (*n*_control_ = 62, *n*_treated_ = 127, *p* = 0.14, rank sum test) (Fig. 7c) or auditory evoked activity between two animal groups (*n*_control_ = 62, *n*_treated_ = 127, *p* > 0.3 across all intensities, rank sum test with Bonferroni correction) (Fig. 7e). We also observed no significant differences across MGB subdivisions (Fig. S3e–h).

We also examined MGB activity in task-performing mice (Fig. 8a). We did not find any significant difference in spontaneous (*n*_control_ = 21, *n*_treated_ = 30, *p*_hit_ = 0.56, *p*_miss_ = 0.90, rank sum test) (Fig. 8b) or auditory evoked activity between animal groups (*n*_control_ = 21, *n*_treated_ = 30, *p* > 0.05, across all intensities, rank sum test with Bonferroni correction) (Fig. 8c). These results imply cortical mechanisms for the hypofunction of auditory processing in XAV939-treated mice.

### Reduced effective excitatory connections in XAV939-treated auditory cortex

To explore a potential mechanism to explain abnormal auditory cortical processing in XAV939-treated animals, we inferred monosynaptic connections among simultaneously recorded neurons by computing cross-correlograms (CCGs) (Fig. 9). This approach allows us to identify strong directional coupling (‘effective connections’) between neurons based on their spike trains^35^. As shown in Fig. 9a, there are three types. Firstly, if there is no strong coupling, no significant modulation can be seen. On the other hand, if there are strong effective connections from one neuron to another, significant excitatory or inhibitory modulations can be seen within several milliseconds after spikes. We systematically assessed these effective couplings across all simultaneously recorded neurons in the auditory cortex (Fig. 9b–d). Across datasets, such connections were generally sparse in both groups (Fig. 9b). However, when we compared the connection probability of individual neurons between two animal groups (18 recordings across 12 control mice, and 26 recordings across 16 treated mice, after exclusion), we found significantly lower connection probabilities in XAV939-treated mice from BS to BS neurons (*n*_control_ = 129, *n*_treated_ = 340, *p* < 0.01, rank sum test with Bonferroni correction) (Fig. 9c). We also noticed a trend toward a reduced connection probability coming from NS neurons, although this was not statistically significant (*n*_control_ = 42, *n*_treated_ = 120, *p* > 0.05, rank sum test with Bonferroni correction) (Fig. 9d). To find if these reductions are layer-specific, as well as look into a possible explanation for differences in laminar cell number and laminar neuronal activity changes, we investigated intra- and interlaminar functional connectivity for BS and NS neurons (Fig. 9e). In XAV939 mice, we observed a significant reduction in excitatory functional projections from L_d1_ to L_d2_ BS neurons (*n*_control_ = 23, *n*_treated_ = 33, *p* < 0.05, rank sum test with Bonferroni correction), as well as between L_d2_ BS neurons (*n*_control_ = 8, *n*_treated_ = 12, *p* < 0.01, rank sum test with Bonferroni correction). We also found a significant reduction in inhibitory functional connections from L_in_ to L_sup_ BS neurons (*n*_control_ = 5, *n*_treated_ = 17, *p* < 0.01, rank sum test with Bonferroni correction). Therefore, broad-to-broad excitatory reduction occurs mainly within deep layers, while L_sup_ receives less inhibitory inputs from L_in_. These results suggest laminar-specific weakened cortical synaptic connections in XAV939-treated mice.

## Discussion

We examined how manipulating the number of superficial cortical excitatory neurons across the brain during embryonic development in mice affects auditory cortical processing in adulthood. We found that XAV939-treated mice exhibit a reduction in behavioral performance during the auditory detection task, as well as having reduced spontaneous activity in BS neurons of the auditory cortex. In addition, the putative monosynaptic excitatory connections between BS neurons were weakened. On the other hand, although auditory thalamocortical neurons exhibited abnormal beta oscillations during silence, MGB neurons could be driven by auditory signals equally between animal groups. Altogether, these results indicate that developmental overproduction of cortical superficial excitatory neurons leads to the hypofunction of auditory processing associated with multiple pathophysiological changes.

A previous report did not find any noticeable effects of XAV939 beyond the cortex^30^. This is probably because neurogenesis in other regions ends before E14.5 when XAV939 was injected. In the mouse auditory pathway, in particular, the peak of neurogenesis in the cochlear nucleus and MGB is E12 and E11, respectively^36^. The superior olivary complex neurons and lateral lemniscus are peaked between E9-13 and E9-12, respectively^37^, while midbrain neurons primarily develop between E11-13 ^36,38^. In addition to this developmental timeline, cellular Axin levels and injection location are likely to be critical factors in restricting XAV939 effects.

Although XAV939 has been used to increase cortical superficial neurons previously, effects of XAV939 microinjections on adult behaviors are varied^30,31^: XAV939-treated mice exhibited autistic behaviors, such as repetitive behaviors and social deficits^30^, and their visual discrimination ability was enhanced^31^. Unexpectedly, we observed a reduced detection ability in the auditory system. This contradiction can be explained by several factors. Firstly, it is possible that different modalities may exhibit distinct effects. Secondly, the overproduction of cortical superficial neurons may lead to distinct impacts on perceptual detection and discrimination. Because superficial excitatory populations encode sensory information sparsely^39–41^, the overproduction of superficial excitatory neurons may increase a coding space, leading to a better discrimination ability whereas this may not be the case for perceptual detection. Indeed, some ASD subjects exhibit decreased signal detection ability while others report enhanced discrimination of simple stimuli in the visual domain^42,43^. In addition, this apparent contradiction may be analogous to the fact that ASD patients often exhibit superior performance for details of their perceptual world, but this comes at a cost for the global perceptive^32,44^. A limitation of the present study was that we did not directly compare detection and discrimination abilities across sensory modalities. Future studies need to reconcile this discrepancy.

As our finding in licking behavior (Fig. 6), the previous studies in this XAV939 model did not observe any motor deficits^30,31^. These results suggest that longer reaction time can be explained by the hyposensitivity to sounds since reaction time to softer sounds becomes longer in general. However, it is still possible that subcortical areas with undetected abnormalities may influence auditory perceptual detection. For example, a recent study showed that the midbrain-thalamus-premotor cortical pathway plays a critical role in the execution of planned actions^45^. Our study does not exclude the possible involvement of other brain regions in observed behavioral outcomes, but our results demonstrate the importance of the auditory cortex. Since our auditory detection task was not designed to delineate sensory encoding and motor execution periods, it remains to be determined to what extent the deficit in auditory detection can be explained by other circuitries.

Related to this, whether or not the auditory cortex is necessary for sound detection has long been debated: while some studies show that the auditory cortex is not needed for sound detection or discrimination^46–49^, other studies report that auditory cortex inactivation significantly diminishes sound detection^50–53^. Our results support the latter case.

Although our results indicate that auditory-evoked activity in the MGB is comparable between animal groups, our results do not rule out the possibility that a compensatory mechanism plays a role in MGB activity^54^. Another caveat is that extracellular electrophysiological recordings rely on spiking activity without monitoring subthreshold membrane potentials. In the future, systematic examinations across the auditory pathway in both sexes are required. In addition to neuronal underpinnings, although the density of astrocytes and microglia are not affected in this model^30^, it is also important to investigate the contributions of non-neuronal cells in more detail.

In both passive listening and task-behaving conditions, we observed variable effects of XAV939 treatment on BS and NS cell activity. However, spontaneous activity in BS cells was consistently reduced in XAV939-treated animals. These observations are also consistent with the previous findings that XAV939 administration shifts the balance between excitatory and inhibitory synapses as a function of age^30^: the ratio of excitatory to inhibitory synapses in L2/3 in XAV939-treated animals increased at P18, whereas it decreased by P60. Thus, the reduced excitatory tone may explain the reduced spontaneous activity in BS cells. However, the underlying mechanism of these age-related changes is unclear. For example, during postnatal development, programmed cell death plays a prominent role in establishing appropriate numbers of excitatory and inhibitory cortical neurons^55,56^. Moreover, accumulating evidence has also demonstrated postnatal activity-dependent processes which sculpt cortical circuits^57,58^ and can be influenced by non-neuronal cells^59,60^. Thus, the overproduction of cortical superficial excitatory neurons during the prenatal period can trigger various compensatory mechanisms, resulting in reduced cortical excitability in adults.

In the present study, we reported a significant reduction in cortical beta oscillations in the XAV939 model for the first time. Although we did not see any changes in hippocampal LFPs, MGB populations also exhibited a similar reduction in beta power. Two potential mechanisms of beta oscillations have been proposed: a cortical mechanism^61^ and a mechanism of the basal ganglia-thalamus-cortical circuit^62,63^. While our results favor the former mechanism, corticothalamic feedback may also play a role and the relation to the basal ganglia remains to be determined in the future. Interestingly, beta oscillations have been implicated in the maintenance of the current sensorimotor or cognitive state^64^. It would be interesting to investigate the reduced beta power in the auditory thalamocortical circuit in the context of this hypothesis.

As in passive listening conditions, we also observed significantly reduced spontaneous activity of BS neurons before “hit” trials in behaving XAV939-treated animals. Additionally, in treated mice, spontaneous activity before miss trials was significantly higher than before hit. On the other hand, we did not observe any significant differences in evoked responses between animal groups. These results suggest that contrary to the passive listening condition, higher background activity in XAV939-treated mice may lead to behavioral hyposensitivity to acoustic stimuli.

Although the expansion of cortical size is advantageous on the evolutionary timescale^11^, our results are consistent with the notion that abnormal expansion of cortical layers during development can lead to an imbalance of excitation and inhibition in the cortical network, resulting in detrimental effects, which can be seen in various neurodevelopmental disorders^65–67^. Indeed, increased cortical volume and L2 neuron number have been reported in ASD^18,68,69^, and some ASD patients experience hypofunction of sensory systems^32^. Such hypofunction may stem from altered cortical connections, and reestablishing synaptic connections may be a promising therapeutic strategy for ASD.

The overproduction of cortical superficial excitatory neurons during development resulted in weaker effective connections among BS cortical populations in adulthood. We further found laminar-specific reductions in function connectivity within deep layers, as well as between L_in_ and L_sup_. We suggest that these altered connections may link to the decrease in spontaneous and evoked BS population activity, as well as auditory response deficits in the mouse model. Since other neural circuits may also play a role, this hypothesis needs to be tested further in the future. It is known that L2/3 neurons have extensive inputs into L5 ^70^. Although we have not observed any significant changes in the functional connectivity between L2/3 (L_sup_) and L5 (L_d1_), we have seen a reduction in excitatory functional connection between L5 and L6 (L_d2_), as well as within L6 in treated mice. It is possible that during development, various compensatory mechanisms arose to account for the differences in L2/3. One of these mechanisms could also be the observed reduction in inhibitory inputs coming from L4 (L_in_) to L2/3.

Additionally, during typical auditory cortical development, connections between layers appear and disappear. For example, during 2nd postnatal week, L5/6 neurons project heavily to L2/3, which later ceases^71^. Our current mouse model also shifts the ratio of excitatory to inhibitory synapses over age^30^. Therefore, it is difficult to conclude what might be the cause and what a consequence of changes in circuitry and cell layer activity over time.

## Methods

### Animal maintenance and XAV939 administration

We confirm that our study is reported in accordance with ARRIVE guidelines (https://arriveguidelines.org). All animal experiments and procedures were performed in accordance with the United Kingdom Animals (Scientific Procedures) Act of 1986 Home Office regulations and approved by the Home Office (PPL70/8883 and PP0688944) and the University of Strathclyde’s Ethical Committee.

The procedure for *in utero* XAV939 microinjections was described previously^25,30^. All mice were obtained from the university’s animal facility. Pregnant female wild-type C57BL/6 mice (*n* = 32) were housed in a 12:12 h light/dark cycle. On embryonic day 14.5 (E14.5), embryos were injected with 0.15–0.4 µl of 10 µM XAV939 (X3004, Sigma Aldrich) in DMSO solution with 0.05% Fast Green dye (F7258, Sigma Aldrich) (treated litter, *n* = 18 pregnant females) or DMSO with 0.05% Fast Green dye solution (control litter, *n* = 14 pregnant females) in the lateral ventricle (LV). Glass pipettes were back-filled with the solution and placed into the pipette holder of the injector (Pneumatic PicoPump PV820, World Precision Instruments). Pregnant females were anesthetized with isoflurane (1.5–2.5%) and placed on a heating pad (ATC1000, World Precision Instruments) facing upwards throughout the surgery. Their abdominal hair was shaved and their skin was cleaned with an antiseptic. A 2-cm longitudinal incision was made along the abdomen, through the skin and then the muscles. Starting at one end and working towards the other, a portion of the uterus containing ~ 3 embryos was extracted at each time using ring forceps. Warm sterile saline (37–38 °C) was used to keep the uterus moist during the procedure. In each embryo, the LV location was identified under a stereoscope (SZ51, Olympus), as a structure at the front of the embryonic brain. Each embryo was held in place with ring-tip forceps. The pipette was advanced through the uterus wall and thin, cartilaginous, transparent skull into the LV. The solution was injected with 2–3 injector pulses. Since Fast Green was added to the injection solution, the dye filled up the LV to confirm the injection site. After all embryos were injected, the uterus was positioned back in its original place. The muscle was sutured, followed by suturing and then stapling the skin. For analgesia, Carprofen (Rimadyl, 10 ml/kg, 0.01% diluted in water for injection) and Buprenorphine (Vetergesic, 3 ml/kg, 0.1% diluted in sterile saline), were administered to the pregnant female subcutaneously.

Since ASD-related abnormalities are more prominent in males^72^, for electrophysiological and behavioral experiments, only male mice were kept until adulthood (perfused ~ P120) and used for behavioral (*n* = 30) and electrophysiological (*n* = 33) assessments (Fig. S1). For histological assessment, both sexes were used at P2 and P21 (n_P2_ = 21 females and 10 males, n_P21_ = 22 females and 5 males). Except histological analysis (see below), all experiments were performed with an open label simply due to practicality (e.g., in utero microinjections for each pregnant mouse were needed to be either XAV939 or vehicle injection).

### Surgical procedure for experiments in head-fixed condition

The procedure was done as described elsewhere^73,74^. Briefly, ~ 8 weeks old male mice were anesthetized with isoflurane (1–1.5%). To provide analgesia, Lidocaine (2%, 0.1 ml) was administered subcutaneously at the site of the incision and Carprofen (Rimadyl, 10 ml/kg, 0.01% diluted in water for injection) was administered subcutaneously in the back. Five screws (418–7123, RS Components) were implanted in the skull, two in the front (AP + 1.5 mm, ML ± 1.5 mm), two on the cerebellum (AP -6 mm, ML ± 2 mm) and one over the right hemisphere (AP -3 mm, ML 3 mm). The posterior left screw was used as a ground, while the anterior right screw was used for cortical electroencephalogram (EEG) recording. The other screws were implanted for anchoring and stabilization of the head cap. A head-post made of 2 nuts was affixed onto the two frontal skull screws. During this surgery, the location of the future craniotomy site above the left hemisphere was also labelled (2 × 2 mm^2^ at AP -2 mm and ML 4 mm). Dental cement was used to cover the skull screws and surface as well as to secure the head-post in place. After the surgery, mice were single housed in high-roofed cages with ad libitum access to food and water and left to recover for at least 5 days.

### Behavioral experiments

*Apparatus* A custom-made behavioral apparatus was located in a soundproof box (Med Associates Inc). A head-fixed mouse was placed in a restraining tube, with a spout, made from a pipette tip, in front of his mouth. The tip of the spout was aligned with a sensor (PMU24, Panasonic). The spout was connected by tubing to a 10 ml syringe filled with water and secured in a single-syringe infusion pump (AL-1000, World Precision Instruments). The pump was located outside of the box. To present the sounds, a calibrated speaker (ES1, Tucker-Davis Technologies) was placed ~ 15 cm in front of the mouse. Water delivery, sound presentation and sensor information were relayed through NI-DAQ (USB-6343, National Instruments) and controlled by a custom-written LabVIEW program (National Instruments). Broadband white noise was generated digitally (USB-6343, National Instruments) and amplified (System 3 ED1, Tucker-Davis Technologies) before being transmitted to the speaker. Licking was detected when the tongue of the animal crossed the above-mentioned sensor beam. The interruption of the beam changed the sensor output, which was relayed through NI-DAQ and detected by the LabVIEW program, and in turn, the program triggered the water pump.

*Behavioral training* After recovery from surgical procedure, mice were placed into the reverse light/dark cycle and water restriction was initiated a week after the adaptation to the new cycle. The restriction was daily, starting gradually until reaching 0.04 ml/g/day. During the restriction acclimation period of 7 days, mice were also habituated to handling and head fixation. Daily training sessions lasted 1 h, during which mice received water through a water pump as a reward (1–3 µl water drop per pump). Mice were weighed after each session and the remainder of their daily water amount was delivered in the form of hydrogel (70-01-5022, ClearH2O). To accurately control the amount of water each animal receives daily, mice were socially isolated at the start of water restriction. Considering that both treated and control mice were isolated during the same period, we do not expect the isolation to be the cause of behavioral differences observed in our groups. Additionally, the training time needed for this behavioral task was similar in both groups of mice (Fig. 2d). Behavioral training consisted of 3 phases: basic lick training, auditory conditioning and auditory detection.

In the first training phase (Basic lick training), mice were familiarized with the system. Whenever the mouse licked the spout, their tongue would cross the beam of the sensor activating the pump and water would be delivered. After making more than 300 licks in a session, the animal would move on to the next phase.

In the second phase (Auditory conditioning), mice were trained to lick only when a broadband white noise (60 dB SPL) was present. In the sound presentation time window, the animal had to lick at least once to elicit a reward. As soon as the licking occurred, the pump immediately delivered the water, and the sound presentation stopped (Fig. 2b). The reward was one droplet of 1.5–2.5 µl of water, depending on the mouse. Animals that got sated very quickly would get less water per droplet, while animals that needed bigger motivation required larger droplets. At first, the sound was presented for 8 s, followed by 2 s of silence. Mice could get a water reward only if they licked during the sound period. After 50 valid trials, the sound would be reduced by 1 s and silence increased by 1 s. The sound presentation was reduced in this way across sessions until reaching 2 s. If an animal had ≥ 150 licks with ≥ 60% success rate for 2 s sound periods in 2 consecutive sessions, they could move on to the final phase.

In the final phase (Auditory detection task), mice were trained and then later assessed in licking whenever they heard a sound, across different intensities. A broadband white noise (30–70 dB SPL, 10 dB SPL steps) was presented for a maximum of 1 s. Intertrial interval (ITI) in this phase lasted 2–4 s, and both intensities and ITI length were pseudo-randomized across trials. Catch trials were also present in this phase, allowing us to assess how often the mouse engaged randomly. When the difference between the catch trial false alarm rate and 70 dB hit rate was more than 40% in two consecutive sessions, after at least 10 training sessions, the mouse would finish training. Only the last session was used in the analysis of behavioral performance.

### In vivo electrophysiology

Detailed recording procedures have been previously described^73,74^. One day before the recording, mice were briefly anesthetized with isoflurane and a craniotomy was done over the auditory cortex (AC) (see above for the coordinate), on the site marked previously during the head cap surgery. Craniotomy site was covered with a biocompatible sealant (Kwik-Sil, World Precision Instruments). During the recording sessions, Kwik-Sil was removed and phosphate buffer saline (PBS) was used to prevent the brain surface from drying. Neural population activity was recorded in ~ 1 h daily sessions over 2 days. Head-fixed, awake mice were sat in a restraining tube within a soundproof box (Industrial Acoustics Company). Electrophysiological recordings were done with either a 64-channel silicon probe (A4 × 16–6 mm-50-200-177-A64, NeuroNexus Technologies; recorded from AC) or with a Neuropixels 1.0 probe (recorded from AC and MGB), mounted on a manipulator (SM-15 or DMA-1511, Narishige). Probes were inserted perpendicular to the cortical surface (AC recording depth 750–900 μm; MGB recording depth ~ 4000 μm). Signals collected through the silicon probe were amplified and digitized (RHD2132 and RHD2000, Intan Technologies Inc.) and recorded using a custom-written LabVIEW program (National Instruments). Signals collected through the Neuropixels probe were amplified and digitized in the probes integrated circuit and recorded using SpikeGLX (Janelia Research Campus). The recording sessions were typically initiated ~ 0.5–1 h after the probe insertion, to allow for signal stabilization. Probes were labelled with either DiI (D-282, Invitrogen, 10% diluted in ethanol) or CM-DiI (C7001, Invitrogen, 0.1% w/v diluted in ethanol) before insertion, thus allowing us to find the probe track later in histology.

*Sound presentation and behavioral task during electrophysiological recording.* For passive listening experiments, broadband white noise (100–200 repetitions of 100 ms pulses with 1 ms cosine ramps, 10 dB SPL steps, 0–70 dB SPL intensity range presented pseudo-randomly, 500 ms interstimulus interval (ISI)), was generated digitally (sampling rate 97.7 kHz, RZ6 Multi I/O processor, Tucker-Davis Technologies) and transmitted in free-field through a calibrated speaker (ES1, Tucker-Davis Technologies). In behavioral task recordings, broadband white noise was presented for a maximum of 1 s (or until the animal licking), in 30–70dB SPL range with 10 dB SPL steps, and 2–4 s ISI. Sound intensity and ISI were presented pseudo-randomly. Behavioral recording sessions lasted 1 h. A water pump (AL-1000, World Precision Instruments) and sound presentation were controlled by a custom-written LabVIEW program (National Instruments). Licking sensor (PMU24, Panasonic) information was relayed through NI-DAQ (NI PCI-6221, National Instruments).

### Histological analysis

For euthanasia, mice were injected intraperitoneally with a mixture of pentobarbital and lidocaine, and perfused transcardially with PBS followed by 4% paraformaldehyde (PFA) in 0.1 M PBS. Brain tissue was removed and stored in the same fixative overnight at 4 °C, then transferred into 30% sucrose solution for at least 2 days. The tissue was cut into 80 μm coronal sections using a microtome (SM2010R, Leica), stained with DAPI, and observed under an epifluorescent upright microscope (Eclipse E600, Nikon). In adult mouse brain slices, post electrophysiological recordings, DiI/CM-DiI signals were also observed to evaluate the probe track.

### Data analysis

*Histological evaluation of superficial cortical layers.* Width of cortical layers in P2 and P21 mice was measured using Fiji ImageJ on DAPI images taken with 4x magnification. Borders between cortical layers were defined in histological images using the known anatomical differences in cortical layers: cortical L5 cells are larger with lower density compared to L4 and L6 (Fig. 1b-c). Thus, we were able to find clear borders between L4 and L5, as well as L5 and L6. Since we couldn’t define the border between L2/3 and L4 confidently in DAPI-stained images, we grouped L2-4 and assessed it as superficial layers width. Portion of superficial layers in the cortex was calculated using the formula: *W*_*L2−4*_/*W*_*L2−6*_, where *W*_*L2−4*_ and *W*_*L2−6*_ were the width of L2-4 and L2-6, respectively. Measurements of 3 adjacent areas were taken on each brain slice, one brain slice per mouse, and the mean of their ratios was compared to other brain slices. All measurements were done blindly to the treatment and grouped afterwards. In P2 mice, the measurements were taken in the frontal, somatosensory area, while at P21 the measurements were taken in the auditory cortical area.

*Behavioral data.* In each session, mean reaction time and detectability index (*d’*) were calculated for all sound intensities. More specifically, to quantify a mouse’s ability to distinguish signal (sound) and noise (no sound), *d’* was calculated using the formula: *d’* = *Z*(hit rate) - *Z*(false alarm rate), where Z is the inverse of the standard normal cumulative distribution function. The detection threshold of each training session was estimated based on cross-validated logistic regression analysis. A logistic binomial model of the probability of hit as a function of sound intensity was created using MATLAB *fitglm* function. The detection threshold was defined as the sound intensity which reaches 50% hit. This process was repeated with a randomly chosen half of trials and the remaining half, and the threshold was calculated as the mean of them.

*Spike sorting.* All electrophysiological data analysis was performed offline. In silicon probe recordings, spike sorting was performed with Kilosort or Kilosort2 (https://github.com/MouseLand/Kilosort), followed by manual curation with phy2 (https://github.com/cortex-lab/phy). Quality of each cluster was assessed by their Mahalanobis (isolation) distance^75^. In all further assessments, we included only the single units with an isolation distance ≥ 20 and an overall spike rate > 0.1 Hz.

In Neuropixels recordings, spike sorting was performed with Pykilosort (https://github.com/MouseLand/pykilosort). Quality of the clusters recognized as “good” by Pykilosort was assessed through additional metrics: sliding refractory period violation and a noise cutoff estimate^76^. Briefly, the first metric estimated whether each cluster is contaminated by refractory period violations without assuming the refractory period duration. The criterion was set as clusters with < 10% contamination. The second metric estimated to what extent spikes were detected without cutting off by the detection threshold. Computing the amplitude distribution of detected spikes, this metric computed how many standard deviations (SDs) of the low bin falls outside the mean number of spikes in the high quantile. The criterion was set as follows: the value was < 5 SDs and the value of the lowest bin was < 10% of the value of the highest bin. Only the “good” clusters passing all thresholds were considered as single units. Out of these units, those with an overall spike rate > 0.1 Hz were included in further evaluations.

*Cortical cell classification*. Cortical cells were further classified based on their spike waveforms^74^. We set the threshold of the trough-to-peak duration to 0.5 ms to distinguish between broad-spiking (BS) and narrow-spiking (NS) cells. In the analysis of electrophysiological data from task-performing mice, we have only included the recordings of sessions where the mice passed the behavioral performance threshold (difference between the false alarm rate and 70 dB hit rate higher than 40%).

*Probe track estimation.* To estimate which channels belong to which brain region in Neuropixels recordings, we assessed the overall multi-unit activity on each probe channel and cross-compared it with a 3D histological evaluation of the probe’s trajectory made by SHARP-Track (https://github.com/cortex-lab/allenCCF/tree/master/SHARP-Track*).* Combining SHARP-Track information with known brain-area specific activity allowed us to determine which recording channels were located in the auditory cortex and which were in the thalamus. Because SHARP-Track borders are not exact, as they are affected by tissue distortion and tearing during slice fixation, we have calculated the median absolute deviation between our electrophysiological and histological borders. On average, this deviation was 69 μm, which is comparable with a previous report^77^.

To define the MGB borders, we took the following two-step estimation approach: (1) the estimation of the number of channels within MGB based on histological analysis, (2) the refinement of the channel positions based on auditory evoked responses. More specifically, by taking the output of the SHARP-Track analysis described above, we estimated the length of MGB passed by the Neuropixels probe. This length was converted to a corresponding number of electrode channels based on the spacing between electrodes. Secondly, we determined the latency of auditory evoked responses across all thalamic channels. For each channel, cell activity was split into 1 ms bins, filtered using 5 ms Gaussian kernel and normalized by evoked response threshold (2.8 SD from baseline mean, taken from 100 ms before the sound onset across trials). Therefore, all values above 1 were considered a significantly evoked response and latency was determined as the first occurrence of such response. Since most MGB neurons have a short latency of auditory stimuli^78^, we identified a cluster of channels exhibiting short response latency (< 20 ms), and refined the MGB channel positions while maintaining the number of channels estimated by the first step. The channels were then further split into MGB subdivisions (ventral, medial and dorsal) based on SHARP-Track area output (Fig. S3).

*Cortical laminar estimation based on current source density (CSD) analysis.* In silicon probe recordings, broadband signals from each channel were lowpass filtered (800 Hz), downsampled (1 kHz) and spatially smoothed across one top and one bottom neighboring channel. In Neuropixels probe recordings, local filed potential (LFP) signals were lowpass filtered (100 Hz), downsampled (1 kHz) and spatially smoothed across ± 5 channels. Event-related potentials (ERP) were then calculated and filtered using lowpass and highpass Butterworth filter, and CSD was calculated using the formula: CSD = ((*Va* + *Vb*)-2*Vo*)/*d*^2^, where *Vo* is the observed channel, *Va* and *Vb* are its neighboring channels and *d* is the distance between channels (mm). CSD sink channel was set as the top border of input layer (L_in_), which was then counted as depth 0, while the borders of deep layer 1 (L_d1_; putative L5) and deep layer 2 (L_d2_; putative L6) were determined based on layer thickness from previous publications^79,80^. Thus, L_d1_ top border was set at − 150 μm and L_d2_ top border at − 400 μm. Everything above 0 μm was considered as superficial layers (L_sup_; putative L2/3). Out of 22 passive listening Neuropixels recordings, 7 recordings (1 from control and 6 from treated mice) were excluded from further laminar and LFP analysis due to excessive noise levels in LFP channels.

*Spontaneous activity analysis.* In passive listening mice, the spontaneous firing rate of each cell was calculated during a 5 min silent period recorded immediately after the noise presentation period. In the same silent period, Welch’s power spectral density was calculated for five frequency bands (delta [1–4 Hz], theta [4–8 Hz], alpha [8–12 Hz], beta [15–30 Hz] and low gamma [30–48 Hz]) for each channel. The relative power at each band was computed relative to the total power (1–48 Hz). Signals from the silicon probes were first extracted with a lowpass filter (800 Hz) and then downsampled to 1 kHz. Only the channels from one (middle) shank were taken into further analysis. For Neuropixels recordings, only one row of channels was used in LFP analysis. LFP signals were lowpass filtered (50 Hz) and spatially smoothed across ± 2 channels before power spectral density calculation. For estimation of mean power per cortical layer, CSD results were used to split the channels into layers, and the mean relative power per layer was calculated for each recording. For cortical and hippocampal mean power estimation, the power across all channels in the auditory cortex or hippocampus was calculated, and their mean per recording was used in group comparisons. For MGB activity, simultaneously recorded MGB unit activity was aggregated and z-scored as normalized multi-unit activity (MUA) with 1-ms resolution. After applying a Gaussian kernel (3 ms) and a lowpass (< 100 Hz) filter, the relative power across frequency bands was calculated. In task-performing mice, spontaneous activity was estimated from 0.5 s periods before the sound onset. For coherence analysis, the same 5-minute time period was analyzed. Only recordings with simultaneous MGB and auditory cortex data were used (n_control_ = 4, n_treated_ = 8). MATLAB *coherencycpt* function was used for MGB spiking and AC LFP signals, across cortical channels, in 2 s time bins with 50% overlap. Mean coherence was then calculated across cortical channels, and in time. Further, for each frequency range, coherence was calculated as the mean in each frequency band.

*Auditory evoked neuronal activity.* For peri-stimulus time histogram (PSTH), average activity during noise presentation of different intensities was calculated in every 1 ms bin across stimulus conditions. For statistical analysis of evoked neuronal activity, after subtracting pre-stimulus baseline activity (50 ms for passive listening and 500 ms for task-performing conditions), spike counts during the 50 ms time window from the sound onset were used.

*Cross-correlation analysis.* Monosynaptic connections assessment was done as described elsewhere^35^. After computing the original cross-correlograms (CCGs), 1000 surrogate CCGs were created to assess statistical significance. To create each surrogate CCG, spikes were randomly jittered ± 3 ms and a CCG was calculated as a surrogate. Based on these surrogate CCGs, upper and lower bounds of confidence limit (1%; *p* = 0.01) were determined. When the original CCG values passed the confidence limit within the [-3:0 ms, 0:3 ms] time window, the interaction between cells was considered significant (*p* < 0.01). If the values passed the upper confidence limit, the interaction between neurons was considered as excitatory. Otherwise, if the values passed the lower confidence limit, the interaction was labelled as inhibitory. The connection probability of individual neurons was calculated as the probability of detecting certain connections given the total number of simultaneously recorded neurons. Depending on connection types (e.g., BS to BS), the connection probability was computed for each neuron. Recordings without at least 2 simultaneously recorded “good” cells were excluded from this analysis.

### Statistics and reproducibility

All statistical analysis was done using MATLAB. The effect was considered significant at *p* < 0.05. Shapiro–Wilk normality test was performed across data, and an appropriate statistical test was chosen based on the results. In Figs. 1d, e, f and g and 2d, S5a and S5c two-sample *t*-test was performed. In Figs. 2e and f and 6b and d, S4 and S5b a two-way ANOVA was performed. Rank sum test was done in Figs. 3c and e, 4c, e and f, 6f, 7c and 8b, S2, S3e and S6. Rank sum test with Bonferroni correction was performed in Figs. 5c, d, e and f, 6g, h, i and j, 7e, 8c and 9c, d and e, S3f, S3g and S3h. To estimate effect size, Hedge’s *g* was calculated in Fig. 4d and S5c, using a MATLAB toolbox^81^.

To balance the sample sizes in spontaneous and auditory evoked passive listening recordings, we reassessed the statistical significance through resampling. We resampled a vector of BS and NS neurons from treated mice of the same size as their control counterparts. This resampling was done 1000 times, and their spike rate (or normalized spike rate) median was assessed each time (Fig. S7, Table S1).

Sample sizes and number of replicates are provided in the figure legends as well as within the main text for each analysis.

## Electronic supplementary material

Below is the link to the electronic supplementary material.

Supplementary Material 1

## Data Availability

The datasets generated and/or analyzed during the current study are available at 10.15129/0e73156e-107c-415d-a648-a3f5ad3554ac.
