# Supplementary material for: Developmental overproduction of cortical superficial neurons impairs adult auditory cortical processing
Source: Sci Rep. 2025 Apr 8;15:11993. doi: 10.1038/s41598-025-95968-x (PMC11978756; doi:10.1038/s41598-025-95968-x)
Supplement: Supplementary file 1 — Supplementary Material 1 [file 41598_2025_95968_MOESM1_ESM.docx]

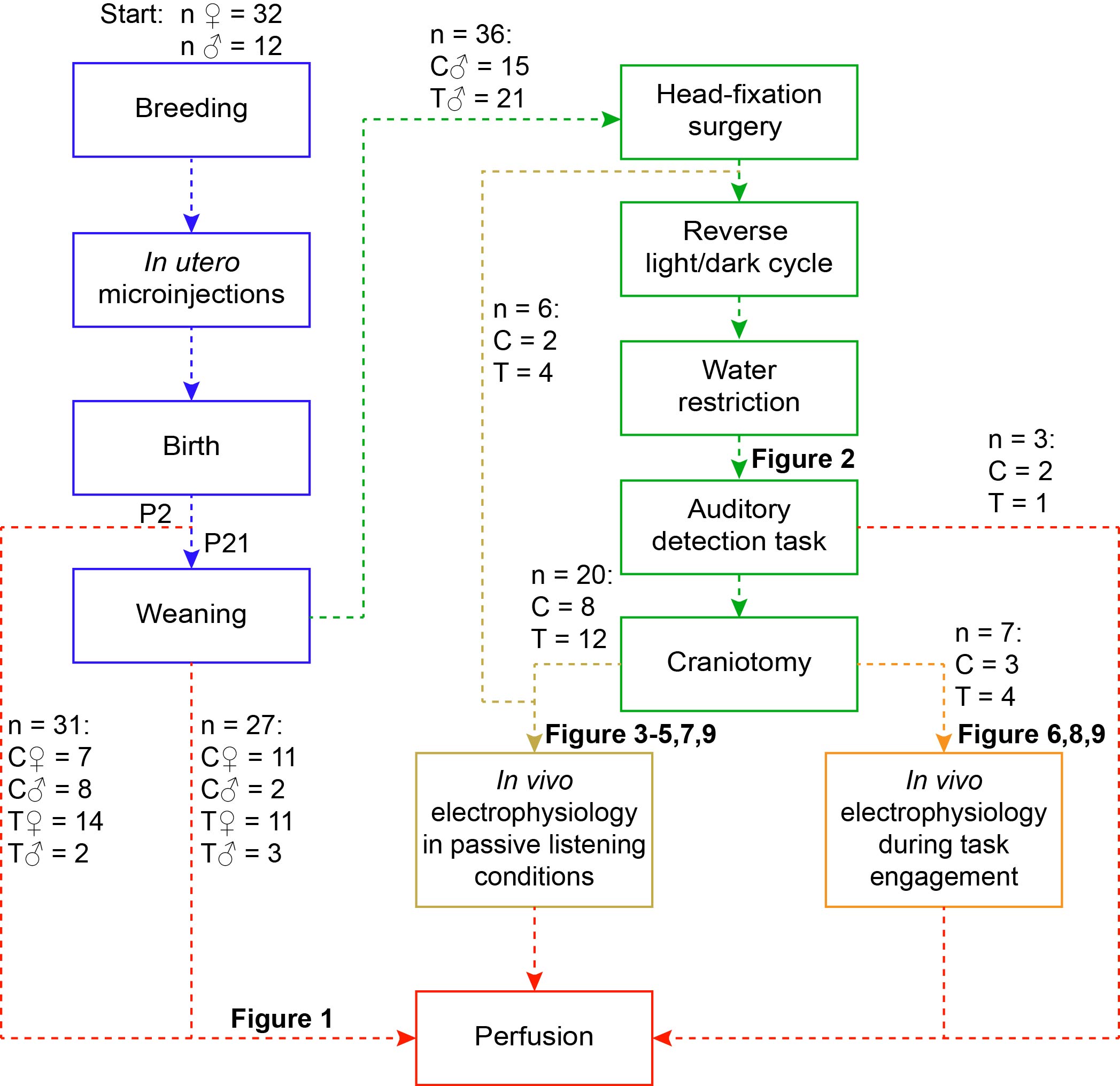


**Figure S1 – Flowchart of the procedures in the project.**

Each male used for breeding was paired with ~3 females. Male breeders were culled afterwards. All pregnant females went through *in utero* microinjections and were culled after weaning the pups on postnatal day 21 (P21). From each litter, a maximum of 2 male pups were kept for further experiments, and the rest of the litter was perfused on either P2 or P21 and used for histological measurements of cortical layers width. Head-fixation surgery was done on the male mice when they were ~8 weeks old. After 2 weeks of recovery and adaptation to reverse light/dark cycle, mice were water restricted and habituated for behavioural assessment. Following successful completion of the task, mice were habituated for electrophysiological recordings. A craniotomy was done and electrophysiological recordings were performed for 2 consecutive days. Mice were perfused afterwards. Several mice which did not finish the behavioural task due to various issues (n = 3), were not used for electrophysiological recordings. Additionally, some mice (n = 6) were recorded without behaviour training, as they were added later to increase the sample size.


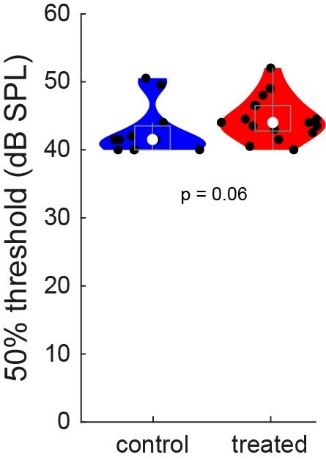


**Figure S2 – Behavioral hearing threshold estimation.**

Detection threshold comparison between control and treated mice (*n* = 11 vs. 16 mice; 1 session per mouse). *p* = 0.06; rank sum test.


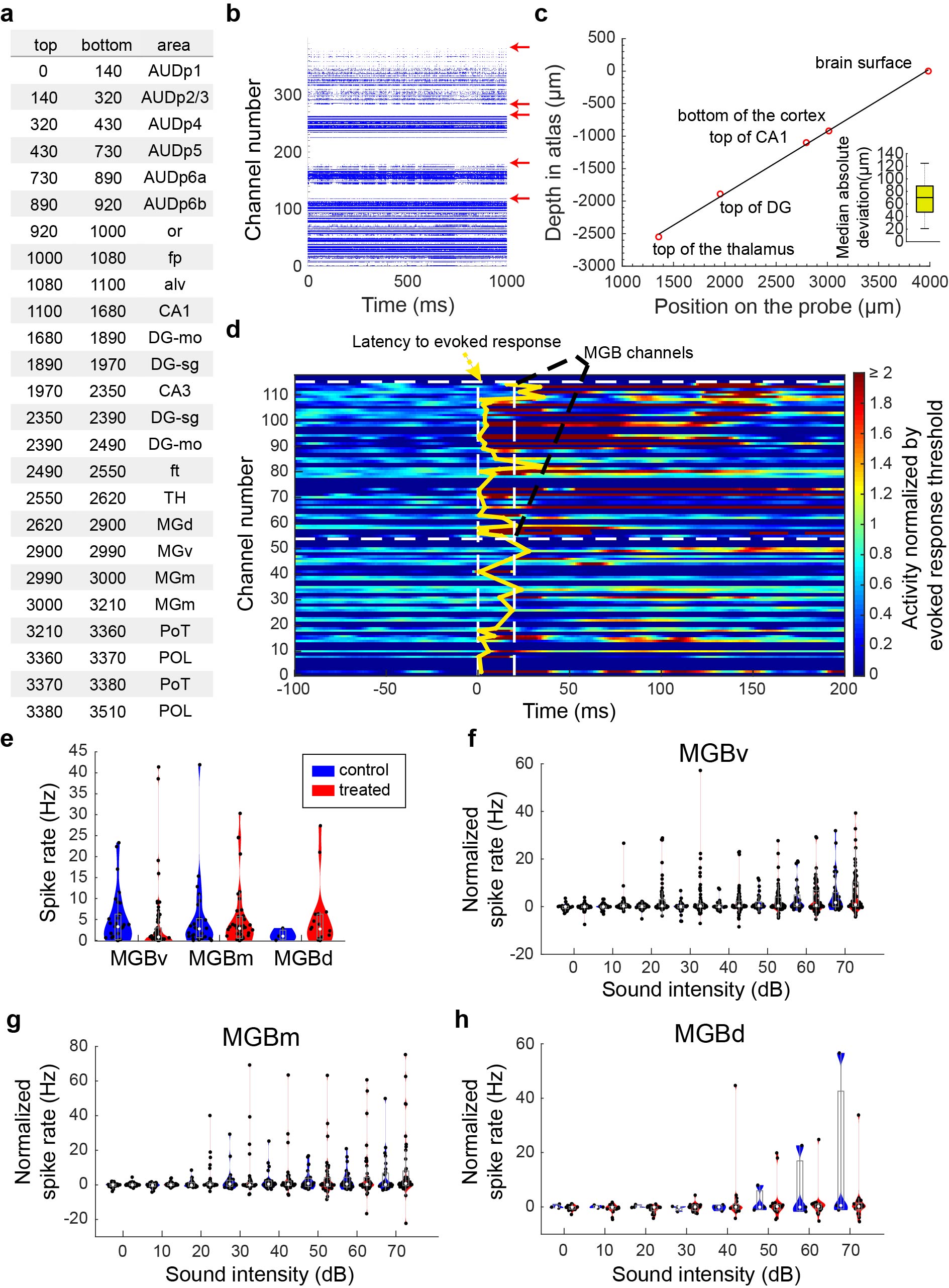


**Figure S3 – Brain area registration and MGB estimation.**

**(a)** Example SHARP-Track output information table containing brain areas penetrated by the probe, and their length in µm on the recording probe. **(b)** Scatter plot of 1 second of neuronal activity across Neuropixels probe recording channels. Using this scatterplot, and histological information shown in **(a)**, we could determine five borders (labelled with red arrows): brain surface, bottom of the cortex, top of CA1, top of dentate gyrus, and top of the thalamus. **(c)** By matching up the 5 borders location between histological **(a)** and electrophysiological **(b)** data, we could assess the median absolute deviation (MAD) between them. Average MAD across all our recordings was 69 µm (inset). **(d)** Because of this deviation, we also used latency to auditory evoked response to refine MGB borders. The onset of the detected evoked response is indicated by a yellow line, across channels. Cluster of channels with short response latency (<20 ms) was identified, and MGB channel positions were refined according to this information. We kept the number of MGB channels (size of MGB) equal to SHARP-Track output information **(a)**. In this example, the total MGB size is 590 µm. We further split the identified MGB channels into subregions based on SHARP Track output information (MGBd 280 µm = 28 channels, MGBv 90 µm = 9 channels, MGBm 220 µm = 22 channels). **(e)** Comparison of spontaneous activity in MGB subregions between control and treated mice (*n*_MGBv_ = 32 vs. 66, *p* = 0.10; *n*_MGBm_ = 27 vs. 42, *p* = 0.85; *n*_MGBd_ = 3 vs. 17 cells, *p* = 0.39, rank sum test). **(f-h)** MGB subregional activity in passive listening conditions across sound intensities. There were no significant differences in neuronal activity between control and treated mice across MGB subregions (*p* > 0.1 across intensities and subregions, rank sum test with Bonferroni correction).


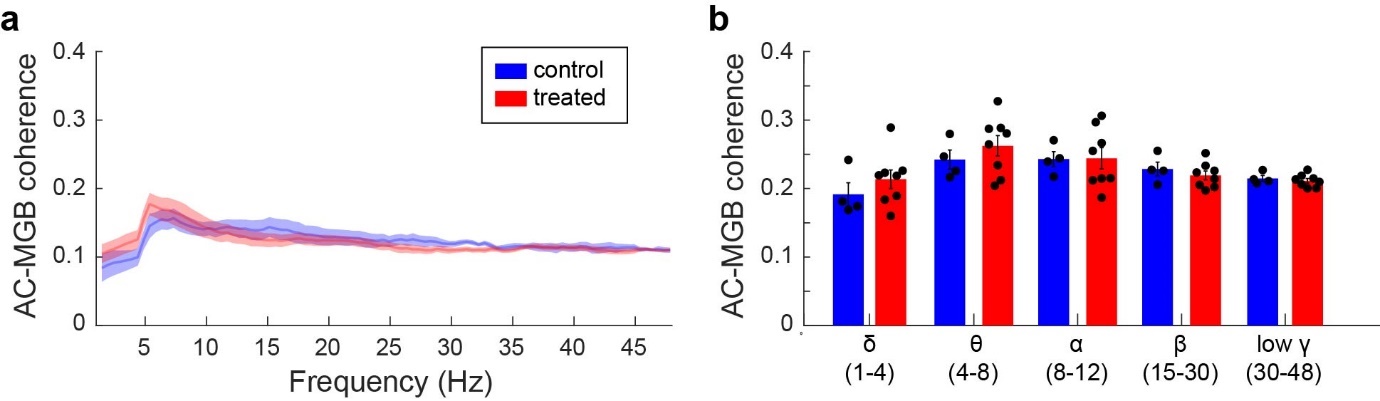


**Figure S4 – Cortical LFP and MGB spiking activity coherence.**

**(a)** Oscillatory coherence between auditory cortical LFP and MGB spiking activity across frequencies (shown as mean ± SEM per treatment group), and grouped in frequency bands **(b)**. There were no significant differences between the treated and control groups (*F*_1,50_ = 0.54, *p* = 0.47, two-way ANOVA; *n* = 4 vs. 8 mice).


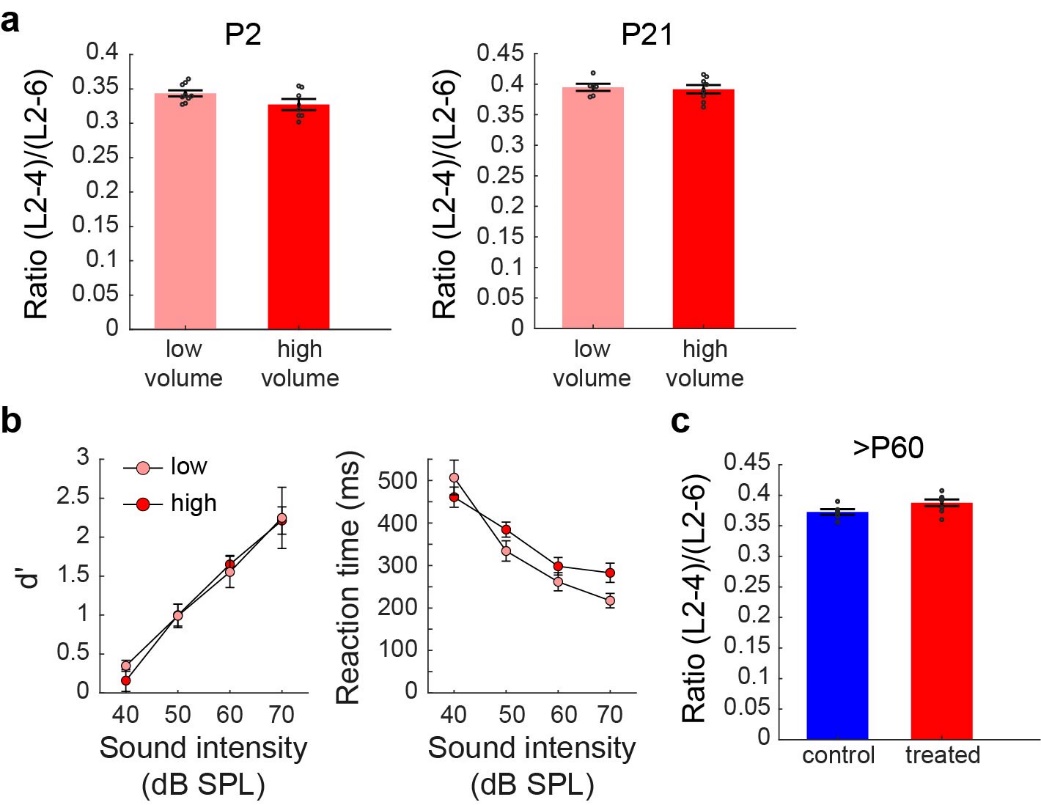


**Figure S5 – Effects of XAV939 volume and animal’s age on superficial layers width.**

**(a)** Comparison of XAV939 volume effect on superficial layers width at P2 (left; *n* = 9 vs. 7 mice) and P21 (right; *n* = 6 vs. 8 mice). We found no significant differences between two volume groups (P2 *p* = 0.1 and P21 *p* = 0.7, *t*-test). **(b)** Comparison of XAV939 volume effect on behavioral d’ (left) and reaction time (right) (*n* = 6 vs. 10 mice). None were significant (d’ *F*_1,56_ = 42.47, *p* = 0.8, reaction time *F*_1,56_ = 36.12, *p* = 0.1, two-way ANOVA). **(c)** Superficial layers width assessment in adult mice (>P60). The difference between control and treated group is not significant (*p* = 0.06, *t*-test), however, the effect size is large (-1.03, Hedge’s *g*; *n* = 6 vs. 8 mice).


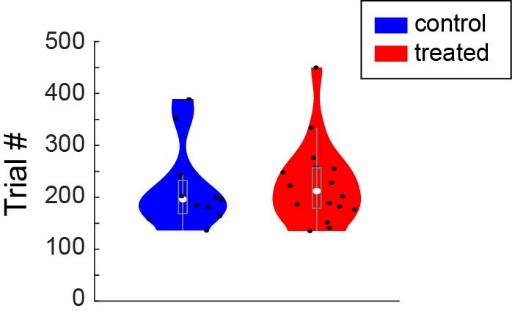


**Figure S6 – Number of behavioral trials across animals.**

Comparison between control and treated group in the number of trials during behavioral session (*n* = 11 vs. 16 mice; 1 session per mouse). The difference is not significant (*p* = 0.62, rank sum test).


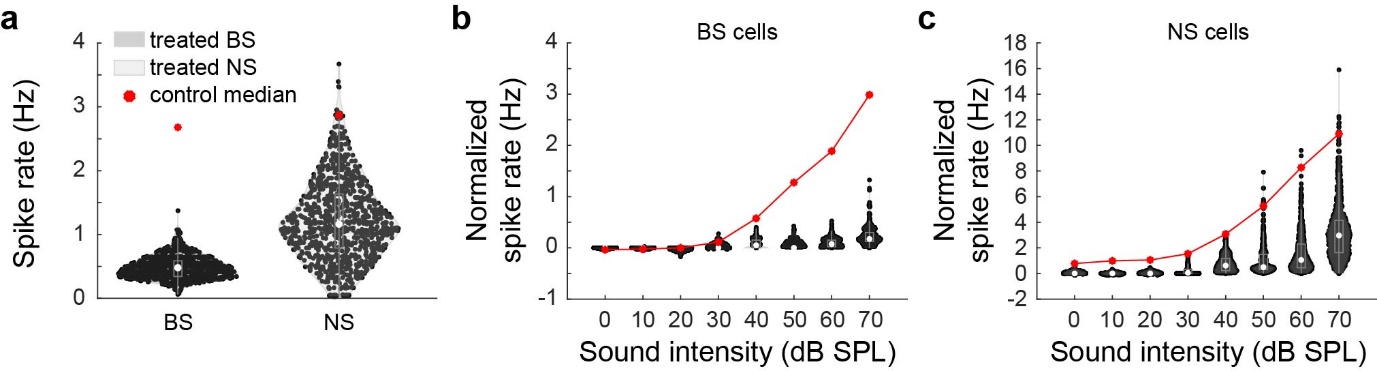


**Figure S7 – Sample size balance.**

**(a-c)** Medians of one thousand times resampled vectors of broad and narrow spiking neurons from treated mice, of the same size as their control counterparts. **(a)** Comparison of resampled treated broad-spiking (BS) and narrow-spiking (NS) spontaneous activity distribution (violin plots) and their control counterpart medians (red point). **(b-c)** Same as **(a)**, for passive listening conditions, across intensities. Probabilities are shown in Table S1.

**Table S1. Probabilities after resampling in Figure S7.**

Probabilities for distributions overlap shown in Figure S7. Columns show conditions (spontaneous or evoked, for BS or NS neurons). In evoked columns, probabilities are further shown for each sound intensity level. Across conditions, almost all probabilities for distributions overlap are significantly low (< 0.05), except for evoked broad spiking 0-20 dB, and evoked narrow spiking 0 dB and 30 dB.

| **Spontaneous broad spiking** | **Spontaneous narrow spiking** | **Evoked broad spiking** | **Evoked narrow spiking** |
| --- | --- | --- | --- |
| 0.00 | 0.02 | 0 dB: 0.99  10 dB: 0.99  20 dB: 0.61  30 dB: 0.02  40 dB: 0.00  50 dB: 0.00  60 dB: 0.00  70 dB: 0.00 | 0 dB: 1.00  10 dB: 0.01  20 dB: 0.02  30 dB: 0.09  40 dB: 0.03  50 dB: 0.01  60 dB: 0.01  70 dB: 0.01 |
